# Why the day is 24 hours long: The history of Earth’s atmospheric thermal tide, composition, and mean temperature

**DOI:** 10.1126/sciadv.add2499

**Published:** 2023-07-05

**Authors:** Hanbo Wu, Norman Murray, Kristen Menou, Christopher Lee, Jeremy Leconte

**Affiliations:** ^1^ Canadian Institute for Theoretical Astrophysics, University of Toronto, 60 St. George Street, Toronto, Ontario M5S 3H8, Canada.; ^2^ Department of Physics, University of Toronto, 60 St. George Street, Toronto, Ontario M5S 1A7, Canada.; ^3^ David A. Dunlap Department of Astronomy and Astrophysics, University of Toronto, 50 St. George Street, Toronto, Ontario M5S 3H4, Canada.; ^4^ Physics and Astrophysics Group, Department of Physical & Environmental Sciences, University of Toronto Scarborough, 1265 Military Trail, Toronto, Ontario M1C 1A4, Canada.; ^5^ Laboratoire d’astrophysique de Bordeaux, Univ. Bordeaux, CNRS, B18N, Allée Geoffroy Saint-Hilaire, F-33615 Pessac, France.

## Abstract

The Sun drives a semidiurnal (12-hour) thermal tide in Earth’s atmosphere. Zahnle and Walker suggested that an atmospheric oscillation with period *P*_res_ ≈ 10.5 hours resonated with the Solar driving ≈600 million years ago (Ma), when the length of day (lod) was ≈21 hours. They argued that the enhanced torque balanced the Lunar tidal torque, fixing the lod. We explore this hypothesis using two different global circulation models (GCMs), finding *P*_res_ = 11.4 and 11.5 hours today, in excellent agreement with a recent measurement. We quantify the relation between *P*_res_, mean surface temperature $\bar{T}$, composition, and Solar luminosity. We use geologic data, a dynamical model, and a Monte Carlo sampler to find possible histories for the Earth-Moon system. In the most likely model, the lod was fixed at ≈19.5 hours between 2200 and 600 Ma ago, with sustained high $\bar{T}$ and an increase in the angular momentum *L*_EM_ of the Earth-Moon system of ≈5%.

## Teaser

The Solar thermal tide drove a resonant atmospheric mode, possibly fixing the length of day at 19.5 hours for a billion years.

## INTRODUCTION

The evolution of the spin of Earth, quantified by the angular momentum *S*_⊕_ [or the length of day (lod)], and the evolution of the orbital angular momentum *L*_☾_ (or semimajor axis *a*_☾_) of the Moon are tightly coupled by gravitational tides, both ocean tides and (much smaller) solid body tides in Earth. The Lunar tide acts to increase both the lod and *a*_☾_, leaving the sum *L*_EM_ = *S*_⊕_ + *L*_☾_ unchanged. The Solar torque −*T*_⊙_ associated with the Solar gravitational tide increases both the lod and the length of the year; it also reduces, very slightly, *S*_⊕_, increasing the orbital angular momentum *L*_⊕_ of Earth by the same amount. Geologic data provide estimates of the lod and the length of the Lunar month (related to *a*_☾_); these data show that the fraction of ocean tidal energy dissipated per Lunar day was lower in the past than it is today.

There are also tides in Earth’s atmosphere, the largest of which are known as thermal tides, as they are driven by sunlight. Thermal tides affect the lod, acting to decrease it, a fact first noted by Thomson (*1*). Thomson focused on the Sun-synchronized semidiurnal thermal tide, as it had the largest observed amplitude at the surface of Earth and had a shape and a phase such that it coupled well to the Solar gravitational tidal field, yielding the largest torque of all the thermal tides. Thomson also suggested that the 12-hour forcing might be near the period of the westward traveling free oscillation with two maxima in Earth’s atmosphere; if true, then the near-resonance might explain why the semidiurnal thermal tide was larger than the diurnal thermal tide, despite the fact that the diurnal temperature variation is larger than the semidiurnal temperature variation. While there is a mode of the type Thomson envisioned, it has long been known that its period *P*_res_ is too far from 12 hours to explain why the semidiurnal tide is stronger than the diurnal tide. We show below that *P*_res_ is closer to 12 hours than previously thought, but the resonance is not responsible for the unexpectedly large semidiurnal torque amplitude.

Zahnle and Walker (*2*) followed up on the idea of a resonance between the thermal forcing and the westward, symmetric, *s* = 2 (meaning semidiurnal) atmospheric mode with a different application in mind. They used a semianalytic one-dimensional model to estimate that the mode had a period of *P*_res_ ≈ 10.5 hours. They suggested that in the past, when the lod was shorter, the mode was indeed resonantly driven, resulting in a much larger thermal tide and tidal torque. They then argued that this would result in a balance between the Lunar tidal torque and the thermal tidal torque, fixing the lod just longer than twice the resonant value of *P*_res_ = 10.5 hours, i.e., a 21-hour day length (lod_res_ = 21 hours).

**Fig. 1.**
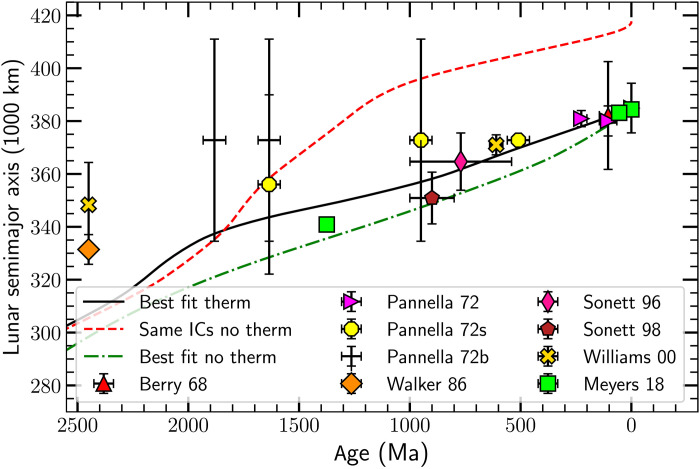
The Lunar semimajor axis as a function of age. The data points are from fossils (*8*–*10*), tidal rhythmites (*11*–*15*), and cyclostratigraphy (*17*). Three curves show the results of our dynamical modeling. The solid black line is the best-fit full (both thermal and gravitational) tide model. The dashed red line shows the result of using the same initial conditions as the best-fit full model but setting the thermal tide to zero; this is not a best-fit model. The dash-dotted green line is the best-fit gravitational tide only model. All models are subject to the constraint that the Moon formed between 4560 and 4510 to 4460 Ma ago, the latter two values indicated by the ages of the oldest known Moon rocks (*23*, *24*), which is why they converge toward each other in the distant past. The models are also constrained by the data for the lod and for the number of days per month (data shown in Figs. 2 and 3). The model runs from the best-fit full-tide initial conditions but neglecting thermal tides (depicted by the dashed red line and labeled "Same ICs no therm") predicts a very large mean Earth-Moon distance *a*_☾_ at the present epoch, the result of a highly dissipative ocean tide at low frequencies in the ocean tidal model (*6*) we use; the low tidal frequencies arise because the model assumes that there is no angular momentum input from thermal tides, so *L*_EM_ is below the currently observed value.

**Fig. 2.**
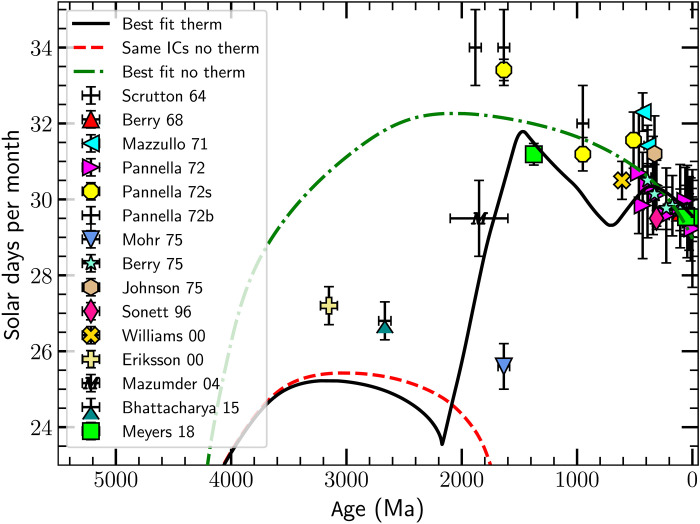
The number of Solar days per Lunar synodic month as a function of age. Data points from (*8*–*11*, *15*, *17*, *25*–*32*). As in Fig. 1, the solid black line is the best-fit model including thermal tides, and the dashed red line shows the result of using the same initial condition but setting *T*_th_ = 0, while the dash-dotted green line shows the best-fit model when *T*_th_ = 0. The tidal rhythmite data older than 1900 Ma (*25*–*27*) lie between the two curves (dash-dotted green and dashed red) for which *L*_EM_ ≈ const., indicating that those data points correspond to states of the Earth-Moon system with different values of *L*_EM_.

**Fig. 3.**
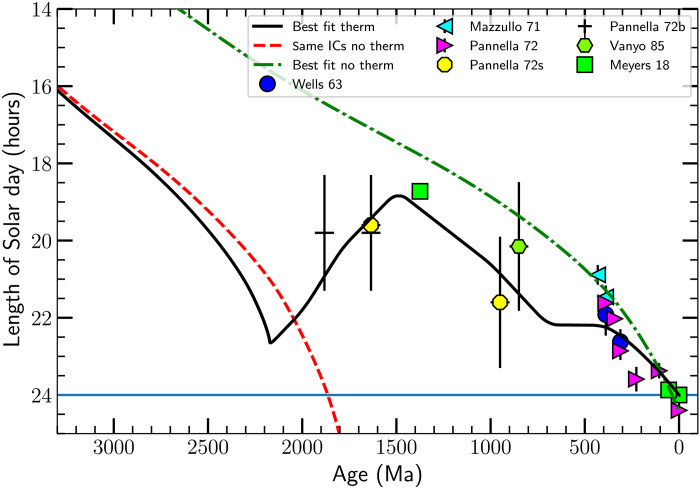
The number of hours per day versus age. The data points are from direct counts (*9*, *10*, *29*, *33*, *34*), except for the green squares, which represent the inferred lod, assuming that *L*_EM_ is fixed at the present day value (*17*). The data points between ≈2000 and ≈1000 Ma suggest that the lod was roughly constant, at about 19 to 20 hours, over that period. The solid horizontal line shows the current lod, 24 hours per Solar day, by definition. The line styles for the three tidal models are as in Fig. 1. The model using the initial conditions for the best-fit full tidal model but setting the thermal tidal torque to zero, indicated by the dashed red line, predicts a present epoch lod of 65 hours, suggesting that the current length of the day was set by the thermal tide. The dashed red line and dash-dotted green lines correspond to nearly constant *L*_EM_, so the data points earlier than ≈1000 Ma are consistent with a smaller initial *L*_EM_. The increase in *L*_EM_ in the best-fit full model (the solid black line) was completed by ≈500 Ma.

We explore and extend Zahnle and Walker’s tidal resonance hypothesis. First, we present geologic evidence from the literature measuring the number of months per year (or *a*_☾_), the number of days per month, and the number of days per year (or the lod, since the length of the year is very nearly constant over geologic time). The results are shown in Figs. 1 to 3 below. The data from epochs earlier than about 1500 Ma ago are very sparse and quite possibly unreliable. What data are available do suggest that the lod was roughly fixed for about a billion years and that the number of days per month increased very rapidly between 2600 and 1500 Ma; these two separate and independent datasets hint that during this time *L*_EM_ increased by about 5%.

We then describe a very simple gravitational tide model, including both the Lunar and Solar tides, together with a simple model for the thermal tide, to predict the evolution of the angular momenta of Earth’s orbit *L*_⊕_, Earth’s spin *S*_⊕_, and the Moon’s orbit *L*_☾_. This allows us to predict the three geologic observables, the number of months per year, the number of days per month, and the number of days per year. Lunar tides, whether ocean, atmospheric, or solid body, do not affect *L*_EM_, while Solar gravitational tides make small (∼1%) changes in *L*_EM_ over geologic time. Hence, any larger variation in *L*_EM_ can be taken as evidence that thermal tides were relevant in the past, which is the question we wish to address here.

Our focus is not on testing gravitational tidal models; a first-principles test of these would require knowledge of the location, shape, and depth of the oceans over geologic time, which, despite recent progress (*3*–*5*), will likely remain out of reach for some time. Rather, we are interested in testing the idea that *L*_EM_ varied over geologic time as a result of thermal tides. Thus, rather than using a sophisticated gravitational tidal model to infer the time at which the Moon formed, we use the known age of the Moon to constrain the specific dissipation rate *Q* in the simple semianalytic tidal model (*6*) we use.

In treating the thermal tides, we take advantage of improvements in atmospheric modeling, going beyond the one-dimensional models used in the past. We use three-dimensional atmospheric models, in the form of two different global circulation models (GCMs), to calculate the period *P*_res_ and amplitude of the gravest symmetric westward wave under current epoch conditions. Our two GCMs find *P*_res_ = 11.4 and 11.5 hours. This period is in good agreement with a recent measurement (*7*) of *P*_res_ = 11.4 ± 0.16 hours. Since the GCMs were in no way tuned to produce the frequencies of this or other normal modes, our result serves to validate the GCMs.

The periods found by the GCMs are notably longer than one-dimensional models find, an important point we return to below. We note that the resonant period is also remarkably close to half the current lod.

We then explore a broad range of Solar luminosities, atmospheric composition (varying the partial pressures of CO_2_ (*P*co_2_) for both GCMS, and O_2_ and CH_4_ for the Laboratoire de Météorologie Dynamique (LMD) model G or Z (which give very similar results), atmospheric mass (or surface pressure), and lod to see how they affect *P*_res_. The Solar luminosity has increased steadily from an initial value 4560 Ma ago of about 70% of its current luminosity. We show that *P*_res_ is primarily sensitive to the global average surface temperature, with a much weaker dependence on atmospheric composition.

We use the geologic data to test, inform, and interpret our models. We use Markov chain Monte Carlo (MCMC) methods to make inferences of the model parameters, yielding best-fit values and confidence ranges. The results include estimates for Earth’s mean surface temperature $\bar{T}$ and for the *P*co_2_, from ∼2200 to ∼600 Ma ago. That is, the combination of GCMs, MCMC methods, dynamical modeling, and geologic data allows us to make inferences on the global climate a billion years ago. We emphasize that, with better data, GCMs can be calibrated against data under conditions very different from those obtaining today; what are now extrapolations to larger CO_2_ levels will become interpolations between today’s level and the much higher levels in the past.

If the thermal tide was in fact dynamically important in the past, then our estimates for $\bar{T}$ serve as a check of isotopic, paleosoil, and other measurements of $\bar{T}$. The estimated CO_2_ levels, which are made at an epoch when few other estimates are available, are near the upper limits found by interpolation from measurements at earlier and later epochs.

Last, if, as the available data suggest, the thermal tide was dynamically important, then the fact that the day is 24 hours long, about twice *P*_res_, is not a coincidence but, instead, results from the transfer of angular momentum from Earth’s orbit to the spin of Earth, via the thermal tidal torque. In the absence of the resonant lock, the current lod would be in excess of 65 hours.

## RESULTS

### Geologic data

#### Synodic months per year

Geologic data give estimates of the number of Lunar months per year (which we convert to Lunar semimajor axis *a*_☾_) with fairly small errors to epochs as far back as 1375 Ma ago and, with far less confidence, to ≈2400 Ma ago (see Fig. 1). Fossil data provide estimates of the number of synodic (or Lunar) months per year (*8*–*10*). Another source of data consists of sedimentary deposits known as tidal rhythmites, recording the number of Lunar months per year (*11*–*13*), or the Lunar nodal precession period (*14*).

We again caution the reader that the early (more than ∼1500 Ma ago) data are of questionable validity. For example, the two data points at 2450 Ma ago in Fig. 1 came from the same patterns in a single tidal rhythmite but interpreted in two different ways; Walker and Zahnle (*14*) interpret them as the signature of Lunar nodal precession, while Williams (*15*) interprets the data as the signature of spring-neap tides in a yearly cycle.

More recently, a second type of analysis, known as cyclostratigraphy (*16*), has been refined and used to measure the lod and *a*_☾_ some 1400 Ma ago (*17*). Cyclostratigraphy relies on the notion that variations in Earth’s surface insolation, driven by changes in Earth’s obliquity, direction of spin, and orbital elements (such as eccentricity and angle of perihelion), are recorded in sedimentary deposits. Interactions with the Moon and with the other planets drive these changes, which are collectively known as Milankovitch cycles (*18*). The causal relation is fairly well established; for example, Hays *et al.* (*19*) measured δ^18^O, the oxygen isotope composition, as a function of depth in sedimentary deposits, combined with absolute ages of some horizons from radiometric dating, to conclude that “changes in Earth’s orbital geometry are the fundamental cause of the succession of Quaternary ice ages.” Note that some authors question this conclusion (*20*).

The physical mechanism driving orbital eccentricity variations is the exchange of angular momentum between planetary orbits; the noncircular, orbit-averaged mass distributions of the planets exert torques on each other, altering each planet’s angular momentum and hence orbital eccentricity and orientation (as quantified by the location of the planet’s closet approach to the Sun or perihelion). These orbit-averaged torques are known as secular interactions. Similarly, interactions between the equatorial bulge of Earth and the orbit-averaged mass distribution of the Moon result in the precession of Earth’s spin. Most of the secular frequencies are known to vary chaotically on 10-Ma time scales (*21*, *22*) and so cannot be used to infer sedimentation rates from the thickness of deposits in the distant past. However, the frequency associated with the difference in perihelion precession rates associated with Venus and Jupiter (denoted by *g*_2_ and *g*_5_, respectively) with *g*_2_ − *g*_5_ corresponding to a period of 405,000 years, is very nearly steady over billions of years. Identification of this period in geologic data allows for researchers to infer the sedimentation rate and, hence, to convert from depth in the sedimentary deposit to elapsed time. This, together with the assumption of a fixed *L*_EM_, allowed a recent measurement of the Earth-Moon distance with subpercent level errors (*17*).

We will argue below that the rhythmite data, as well as, with less confidence, the stromatolite data, suggest that *L*_EM_ varied by about 5% over a billion years, so that future cyclostratigraphic analysis should allow for that possibility. Last, securely dated Moon rocks show that the Lunar surface solidified at least 4460 ± 40 Ma ago (*23*) or even 4510 ± 10 Ma ago (*24*), strong but indirect evidence that Lunar tides were active at least since that time.

Combining all three types of data constrains *a*_☾_(*t*), and, hence, the Lunar orbital angular momentum *L*_☾_(*t*) and the duration of the Lunar month, fairly tightly. We combine these data with data on the number of days per month, as well as the lod, described below, to constrain the evolution of the Earth-Moon system.

The curves in Fig. 1 show a best-fit model with gravitational tides only (the dash-dotted green line) and a best-fit model including both thermal and gravitational tides (the solid black line). The dashed red line shows the result of using the initial conditions from the best-fit full model but then ignoring the thermal tide, i.e., this is not a best-fit model. The models are described below.

#### Days per synodic month

Tidal rhythmites can also record the number of Lunar days per synodic month; these deposits show directly that Lunar tides have been active since at least 3.2 billion years ago (Ga ago) (*25*). Figure 2 shows data from this and more recent epochs (*11*, *15*, *17*, *25*–*27*). The figure also shows data from nontidal rhythmites (*17*) and fossil data (*8*–*10*, *28*–*32*), the latter often recording the number of Solar days per synodic month (in all cases, we report Solar days per synodic month). The tidal rhythmite data indicate that there were about 27 Solar days per synodic month (corresponding to 26 Lunar days per synodic month) around 3000 Ma; this count then increased over ≈1000 Ma to about 31 Solar days per month, followed by a gradual decline over the past 1000 Ma to the present day value of 29.5 Solar days per month. The cyclostratigraphic data (*17*) have smaller error bars than those associated with the stromatolite data (*10*, *30*) and are likely to be far more precise than the latter, but the data point at 1400 Ma assumes constant *L*_EM_, as noted above.

There are undoubtedly systematic errors associated with the tidal rhythmite data. For example, it is plausible that some tides are unrecorded because of inclement weather. How many missing days would there have to be to move the yellow cross [from (*25*)] in Fig. 2 from the nominal value of 27.2 to 28.2 Solar days per month? There are 120 layers (forsets, in their Fig. 3), corresponding to about nine fortnights (4.5 months) at the nominal 13.1 Lunar days per fortnight. (Note that we plot this as 27.2 Solar days per month). If the actual number of Solar days per month was 27.2, then there would have to be 125 forsets. Alternately, the number of fortnights would have to be 8.66 rather than 9.16. Missing 5 forsets of 120 is certainly possible, but missing 10 is unlikely. Similarly, missing a neap tide seems possible, but missing two seems unlikely. This is how we estimated the error bar, with a length of about 1 day per month, in the plot.

Moving the oldest point on the plot up to the best-fit no–thermal tide model would require 134 forsets, i.e., there would have to be 14 unrecorded tides. Similar comments apply to the result of (*27*).

Figure 2 also shows the three models shown in Fig. 1. Anticipating our dynamical results, we note that both the dashed red curve and the dash-dotted green curve in Fig. 2 represent models that, aside from the small effect of Solar gravitational tides, conserve *L*_EM_. As a result, they share similar shapes, with the number of days per month first increasing, smoothly reaching a maximum (at around 3000 Ma for the dashed red curve and 2000 Ma for the dash-dotted green curve) before decreasing smoothly. The shape of the black curve is more complex; it tracks the dashed red line for ages earlier than 3200 Ma, while it tracks the dash-dotted green line for ages later than 400 Ma. Between about 2400 and 400 Ma, the black curve shows markedly different behaviors, with alternating sharp increases and decreases in the number of days per month. These reversals reflect two different resonant states, in which the thermal tidal torque equals or exceeds in magnitude the gravitational tidal torques. During this time, the thermal tide produces an increase in *L*_EM_ by about 5%.

We note that the three tidal rhythmite data points older than 1900 Ma, from (*25*–*27*), lie between the two curves with (nearly) fixed *L*_EM_, hinting that the value of *L*_EM_ may have varied over geologic time.

#### Hours per day

Last, fossil data record the number of days per year or, equivalently, the number of hours per day [bivalves and corals (*9*, *10*, *29*, *33*) and stromatolites (*9*, *10*, *34*)] from direct counts of the number of daily growth ridges per annular pattern (Fig. 3). Figure 3 also shows three green filled squares depicting the lod inferred (by assuming that *L*_EM_ is fixed at the present day value) from cyclostratigraphy (*17*).

The best-fit no–thermal tide model, depicted by the dash-dotted green line in Figs. 1 to 3, and the no–thermal tide model using the initial conditions from the best-fit thermal tide model (the dashed red line in Figs. 1 to 3) conserve the angular momentum *L*_EM_ of the Earth-Moon system at roughly the 1% level. The fact that the data points in Figs. 2 and 3 systematically vary between the two curves suggests that some nontidal torque acted on Earth or the Moon. This non-*L*_EM_ conserving torque is most apparent in the data between 3200 and 1600 Ma in Fig. 2 and between ≈1800 and 800 Ma in Fig. 3. The change in *L*_EM_ is about 5%, substantially larger than and opposite in sign to the change induced by the Solar gravitational tide.

### Gravitational tide model

We are interested in exploring the possible effects of a thermal tide, but we still need to model the gravitational tide. The gravitational tidal torque is proportional to the amount of energy dissipated in the tide. It is generally believed that ratio of energy stored in the tide to the tidal energy dissipated per tidal period (12 hours), denoted by *Q*, is a function of frequency and, further, that *Q* depends on the size, shape, and bathymetry of the oceans. We assume that some combination of changes in tidal frequency (related to the lod) and changes in the oceans must result in slower evolution than the simple fixed *Q* model, but we do not attempt to find a first-principles model that does so.

Instead we choose to use a simple tidal model that neglects the obliquity of Earth as well as the eccentricity and inclination of the Moon’s orbit, in which the Lunar tidal torque is (*35*, *36*)

$$T_{\text{☾}}=\frac{3}{2}\frac{Gm_{\text{☾}}^{2}}{a_{\text{☾}}}{\left(\frac{R_{⊕}}{a_{\text{☾}}}\right)}^{5}\frac{k_{2}}{Q_{1}Q(\omega^{t})}$$ (1)

In this expression, *G* is the Newton’s gravitational constant, *m*_☾_ is the Lunar mass, *R*_⊕_ is the radius of Earth, and *k*_2_ is the (dimensionless) Love number (*35*) of Earth (see table S1, which lists numerical values for relevant quantities). The tidal quality factor *Q* = *Q*_1_*Q*(ω) corresponds to 1/ sin 2δ in (*35*). We treat *Q*(ω) as a known (and fixed, as far as our MCMC calculations are concerned) frequency–dependent function (see fig. S1); the Lunar semidiurnal tidal frequency $\omega_{\text{☾}}^{t}=2({\text{Ω}}_{⊕}-n_{\text{☾}})$, where Ω_⊕_ = 2π/*P*_⊕_ with *P*_⊕_ as the length of the sidereal day. The Lunar mean motion $n_{\text{☾}}\approx \sqrt{G(M_{⊕}+m_{\text{☾}})/a_{\text{☾}}^{3}}$, an expression that neglects the effect of the Sun’s gravity on the Lunar orbit. We neglect the frequency dependence of *k*_2_ and use the present day value *k*_2_ = 0.298. We neglect any changes in *Q*(ω) that might result from changes in the size or shape of the oceans, a fairly marked approximation. However, we allow for the overall normalization of *Q* = *Q*_1_*Q*(ω) to vary in the MCMC runs, by introducing a new variable *Q*_1_ that is of order unity. This is necessary to allow the Moon’s orbit to expand slowly enough to match the know minimum Lunar age.

At the present epoch, *T*_☾_/*T*_th_ ≈ 11.5 (*37*) (see table 6.4 in that reference) and *T*_☾_/*T*_⊙_ ≈ 4.7 (see section S1), so that evolution of Earth’s angular momentum is currently dominated by ocean tides. Laser ranging measurements (*38*) show that the semimajor axis *a*_☾_ of the Moon is increasing at the rate of ${\dot{a}}_{\text{☾}}=3.82\pm 0.07\;\mathrm{cm}/\mathrm{year}$, corresponding to a torque *T*_☾_ ≈ 4.55 × 10^23^ dyne·cm.

While we use a simple gravitational tide model, we emphasize again that the Lunar tides cannot affect *L*_EM_. It follows that any shortcomings in the gravitational tide model we use cannot be responsible for the differences between the data and either of the no–thermal tide models (the dashed red line or the dash-dotted green line) in Figs. 2 and 3.

### Thermal tides

Atmospheric tidal theory has a long history; see (*39*) for a clear presentation. The classical theory linearizes the equations of motion and then assumes a separation of variables of the form (using the perturbed pressure *P* as an example)

$$P(\theta ,\phi ,z,t)=\sum_{n,s,\sigma }L_{n}^{\sigma ,s}(z){\text{Θ}}_{n}^{\sigma ,s}(\theta )\mathrm{ex}p^{i(\sigma t+s\phi )}$$ (2)

where θ is the colatitude, ϕ is the east longitude, *z* is the height above the surface, *s* is an (possibly negative) integer, and σ is the frequency of the oscillation [see equation 24 on p110 of (*39*)]. Substituting this (and similar expressions for the three velocity components, density, etc.) and some algebra results in two separated equations, one known as Laplace’s tidal equation (since he derived it for ocean tides) for Θ(θ) and the second for $L_{n}^{\sigma ,s}(z)$, known as the vertical structure equation, as well as separation constants. The latter are traditionally taken to have units of length, $h_{n}^{\sigma ,s}$. By analogy with Laplace’s theory of ocean tides, they are called equivalent depths; in analytic one-dimensional models, they are directly related to the frequencies of free oscillations. By contrast, it is fairly simple to find resonant frequencies from our GCMs, while it is not so simple to find the $h_{n}^{\sigma ,s}$. Further, recent measurements directly yield the frequencies of free oscillations (*7*), so we generally report the frequencies and use the results of one-dimensional models to convert to the equivalent depths. The functions ${\text{Θ}}_{n}^{\sigma ,s}(\theta )$ are known as Hough functions.

As noted in Introduction, the Sun produces the thermal tide by heating Earth’s surface and atmosphere, driving air away from the highest temperature regions, resulting in bulges in the atmosphere. These bulges produce variations in barometric pressure with a frequency corresponding to a period of 24 hours and harmonics, which are readily apparent in data from the mid-1800’s (*40*); the component with a period of 12 hours produces a significant torque. Using these data and noting that the Sun’s gravity would attract the atmospheric bulges, Thomson (*1*) estimated *T*_☾_/*T*_th_ ≈ 10, close to the value of 11 quoted above. Using the modern estimate gives a present day value of *T*_th_(0) = 4.14 × 10^22^ dyne·cm.

In section S2, we show that the pressure perturbation driven by the Solar atmospheric heating produces a torque

$$T_{\mathrm{th}}=A(t)T_{\mathrm{th}}(0)\frac{f({\tilde{\text{Ω}}}_{⊕},\omega_{\mathrm{th}},Q_{\mathrm{th}})}{f[{\tilde{\text{Ω}}}_{⊕}(0),\omega_{\mathrm{th}}(0),Q_{\mathrm{th}}(0)]}$$ (3)

where *A*(*t*) is a time-dependent normalized torque amplitude and *t* = 0 corresponds to the present (see figs. S2 to S4). We call *A*(*t*) a normalized torque amplitude, since *A* = 1 yields a torque that is equal to the present day atmospheric thermal torque, *T*_th_(0).

We use a simple piece-wise linear model for *A*(*t*)

$$A(t)=\left\{ \begin{array}{cc} A_{1}+(A_{2}-A_{1})(t/t_{0}) & t<t_{0}\\ A_{2} & t\geq t_{0} \end{array}\right.$$ (4)

This introduces three parameters, two that we vary as part of the Monte Carlo calculation (*A*_1_ and *A*_2_) and one that we consider fixed, the age at which the normalization begins to decrease, denoted *t*_0_. Note that *t* runs from 4560 Ma ago to the present (0). We have tried various values 1000 Ma < *t*_0_ < 2500 Ma, so that *A*(*t*) = *A*_2_ in the Hadean and Archean. The results do not depend strongly on the exact value, so we set *t*_0_ = 2000 Ma.

**Fig. 4.**
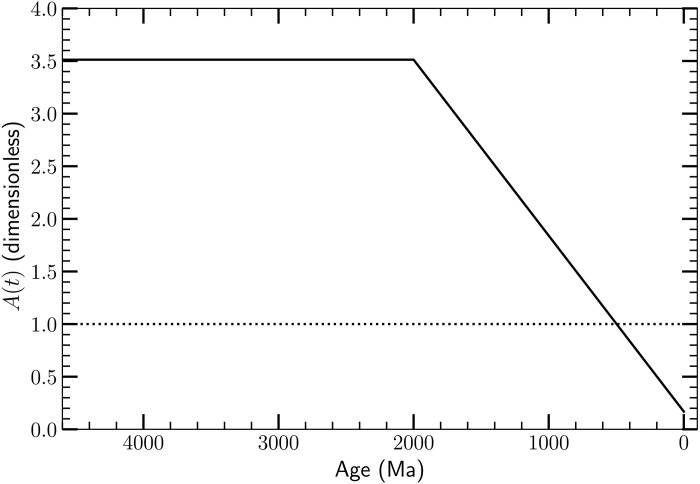
The normalization *A(t*) of the thermal torque versus age for our best-fit full physics model. The horizontal dotted line is the nominal value *A*(*t*) = 1 corresponding to the present-day torque acting on the atmosphere, i.e., not taking into account the possible static (inverse-barometer) response (*90*) of the oceans. If the oceans respond statically to the pressure variations at the ocean surface due to the thermal tide, then that would tend to make *A* < 1. If the atmospheric mass or the *P*co_2_ were higher (as they likely were in the past), then that would tend to make *A* > 1. The low value we infer for *A*(0) may be driven by the data from (*17*), who assume that *L*_EM_ is constant; see the main text.

The dynamical model, when constrained by the data, then prefers *A*_1_ < *A*_2_, i.e., the thermal torque was larger in the past than at present. Figure 4 shows the normalized torque as a function of age for our best-fit thermal tide model. We find *A*_2_/*A*_1_ ≳ 20, an unexpectedly large ratio, which we discuss further below.

The function

$$f({\tilde{\text{Ω}}}_{⊕},\omega_{\mathrm{res}},Q_{\mathrm{res}})\equiv \frac{4{\tilde{\text{Ω}}}_{⊕}({\tilde{\text{Ω}}}_{⊕}^{2}-\omega_{\mathrm{res}}^{2})+{\tilde{\text{Ω}}}_{⊕}\omega_{\mathrm{res}}^{2}/Q_{\mathrm{res}}^{2}}{4{\left({\tilde{\text{Ω}}}_{⊕}^{2}-\omega_{\mathrm{res}}^{2}\right)}^{2}+{\tilde{\text{Ω}}}_{⊕}^{2}\omega_{\mathrm{res}}^{2}/Q_{\mathrm{res}}^{2}}$$ (5)

where the frequency ${\tilde{\text{Ω}}}_{⊕}\equiv 2\pi /\mathrm{lod}$. The frequency ω_res_ = 2π/(2*P*_res_), with *P*_res_ as the period of the atmospheric resonance, measured at a fixed point on the planet. In the “Global circulation models” section, we use our GCMs to estimate that at the present epoch, *P*_res_(0) ≈ 11.4 or 11.5 hours, but the results of our dynamical modeling, constrained by the data, show that *P*_res_(*t*) was possibly as short as ≈9.5 hours. Last, *Q*_res_ is the quality factor of the atmospheric resonance, which we take to be the same at all epochs, although it needs not be.

### Global circulation models

To use Eqs. 3 to 5, we need estimates for the atmospheric resonant period *P*_res_(*t*), the normalized amplitude *A*(*t*), and the quality factor *Q*_res_ of the resonance. Both *P*_res_ and *A*(*t*) depend on the atmospheric composition (which we denote by *Z*), the mean surface pressure ${\bar{P}}_{s}$ (or the mass of the atmosphere), the mean incoming Solar flux $\bar{F}$, and the lod *P*_⊕_. Geologic evidence shows that *Z* varies with time (see section S3), while stellar evolution theory predicts that the Solar luminosity and hence $\bar{F}$ increase with time. Variations in the mean ${\bar{P}}_{s}$ have been suggested, and there are some geologic constraints, but the latter are fairly weak, so in what follows, we explore the effects of changes in ${\bar{P}}_{s}$ as well. We use the notation $\text{Λ}\equiv (\bar{F},Z,{\bar{P}}_{s})$.

We use two GCMs, PlaSim (*41*) and LMD-G (*42*, *43*) (see section S4). LMD-G includes more, and more sophisticated, physics than does PlaSim, but the latter runs much faster. We use PlaSim to explore parameter space, with spot checks from LMD-G to ensure that the results are robust. The GCMs calculate, among other things, the pressure variation in the atmosphere and, in particular, the surface pressure, on a grid of longitude and latitude. Following (*44*), we use the surface pressure perturbation to calculate the complex amplitude of the mass quadrupole (the analog of $P_{2}^{2}$ in the one-dimensional model in eq. S25) by projecting the pressure perturbation onto spherical harmonics and then use their eq. S1 to find the torque *T*_th_.

### Dynamical model

We are now in a position to model the effects of both thermal and gravitational tides on the evolution of the Earth-Moon system. We neglect the spin angular momentum of the Moon since the Moon’s moment of inertial is roughly a thousand times smaller than that of Earth (*45*), leaving three dynamical equations

$$\frac{dL_{⊕}}{dt}=T_{⊙}-T_{\mathrm{th}}$$ (6)

$$\frac{dS_{⊕}}{dt}=T_{\mathrm{th}}-T_{⊙}-T_{\text{☾}}$$ (7)

$$\frac{dL_{\text{☾}}}{dt}=T_{\text{☾}}$$ (8)

The torque *T*_☾_ is given by Eq. 1, *T*_⊙_ by eq. S2, and *T*_th_ by Eqs. 3 to 5. Our sign convention is that both *T*_☾_ and *T*_⊙_ are positive. *T*_th_ can be of either sign; today, it is positive, but for times earlier than ≈2200 Ma, we will show that it was negative.

### Model results

#### GCM results

**Fig. 5.**
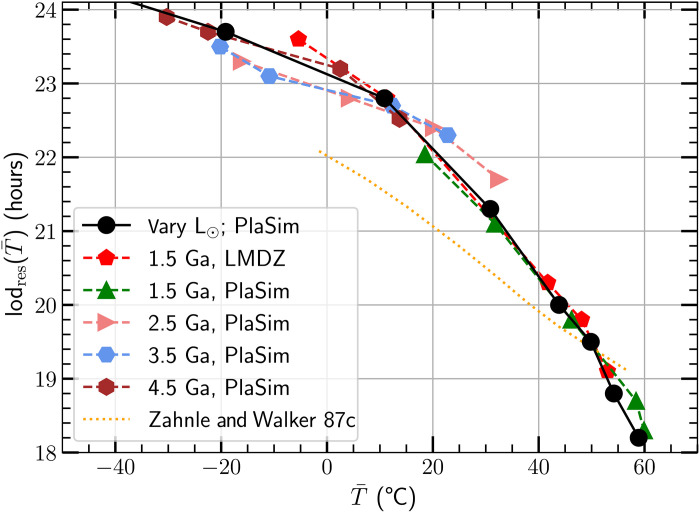
The resonant lod as a function of mean Earth temperature. The green triangles correspond to PlaSim runs with a fixed ${\bar{F}}_{⊙}=0.88{\bar{F}}_{⊙,0}$ (corresponding to an epoch of 1.5 Ga) and a surface pressure *P*_s_ = 2 bar. The points depict *P*co_2_ =[1,10,100,200,300] mbar, with higher *P*co_2_ yielding higher $\bar{T}$. Red pentagons correspond to LMD-G runs at the same epoch, with *P*_s_ = 2 bar. Pink triangles show Plasim results for ${\bar{F}}_{⊙}=0.82{\bar{F}}_{⊙,0}$ (2.5 Ga), light blue hexagons show ${\bar{F}}_{⊙}=0.77{\bar{F}}_{⊙,0}$ (3.5 Ga), and brown hexagons correspond to ${\bar{F}}_{⊙}=0.72{\bar{F}}_{⊙,0}$ (4.5 Ga); for all these the lowest *P*co_2_ =10 mbar. For a given *P*co_2_, LMD-G returns $\bar{T}$ values lower by ∼5 to 10°C or about 2 to 8%, than those found by PlaSim. The black circles depict PlaSim runs with the present epoch atmosphere but with different ${\bar{F}}_{⊙}$. The orange dotted line is taken from the one-dimensional atmospheric model labeled “c” in figure 3 of (*2*), where the temperature is varied directly. Note that, while PlaSim and LMD-G find different relations between $\bar{T}$ and *P*co_2_, the two codes find very nearly the same relationship between $\bar{T}$ and *P*_res_. Furthermore, both codes predict a relation between *P*_res_ and $\bar{T}$ that mimics the one found by PlaSim when holding *P*co_2_ fixed and varying $\bar{F}$.

We define the resonant lod to be twice the resonant period of the westward propagating (*s* = 2) mode *P*_res_ of the atmosphere, i.e., lod_res_ = 2*P*_res_. Figure 5 shows the relationship between lod_res_ and the global mean surface temperature, $\bar{T}(\text{Λ})$, for two GCMs (LMD-G and PlaSim) and for one of the one-dimensional atmospheric models in (*2*). See Materials and Methods for a description of how we determined lod_res_ for our GCMs.

The GCM results for the present epoch are in remarkably good agreement with a recent measurement of the frequency of the westward traveling wave. Table 1 in (*7*) lists the frequency of a number of normal modes, including a frequency of 2.11 ± 0.03 cycles/day, corresponding to a period of 11.4 ± 0.16 hours for the *s* = 2 westward (*k* = −2 in their notation) symmetric mode. PlaSim finds a frequency of 2.097 cycles/day, or *P*_res_ = 11.44 hours. We are unaware of previous work comparing the frequencies of free oscillations in GCMs to observed frequencies, although GCMs have been used to calculate the amplitude and phase of the response to Solar heating, e.g., (*46*, *47*).

The red pentagons (LMD-G) and green triangles (PlaSim) in Fig. 5 show predictions for the atmospheric resonant period $P_{\mathrm{res}}[\bar{F}=0.88\bar{F}(0),Z,P_{s}=2\;\mathrm{bar}]$, where we use the Solar flux at an age of 1.5 Ga (*48*). We set the partial pressure of O_2_ (*P*o_2_) to 0.001 bar, appropriate for that age (*49*, *50*). We also set *P*_s_ = 2 bar; the associated pressure broadening and enhanced greenhouse gas opacity leads to temperatures high enough to produce *P*_res_ as short as 18.5 hours, as implied by the geologic data. From left to right, the triangles and pentagons correspond to *P*co_2_ =[1,10,100,200,300] mbar.

For a given *P*co_2_, PlaSim returns a mean global $\bar{T}$ about 5° to 20°C larger than that found by LMD-G, for an age of 1000 Ma, when the Solar luminosity was 12% lower than today. For *P*co_2_ =[1,10,100,200,300] mbar, we find $\bar{T}={\left[18.5,31.8,46.2,58.4,59.9\right]}^{\circ }C$ for PlaSim and $\bar{T}={\left[-5.4,11.3,41.7,48.1,53.0\right]}^{\circ }C$ for LMD-G. This reflects the different treatment of physics in the two codes. However, for a given $\bar{T}$, the figure shows that the two GCMs return very nearly the same resonant period *P*_res_. The pink triangles in Fig. 5 show PlaSim predictions for $\bar{F}=0.82{\bar{F}}_{⊙}$ (2.5 Ga), and light blue hexagons correspond to $\bar{F}=0.77\bar{F}(0)$ (3.5 Ga), while the brown hexagons are for $\bar{F}=0.72\bar{F}(0)$ (corresponding to 4.5 Ga). For all these, *P*_CO_2__ =[10,100,200,300] mbar.

The filled black circles show the result from PlaSim holding *Z* and *P*_s_ at the current epoch values while varying $\bar{F}$. The relation between *P*_res_ and $\bar{T}$ is very nearly the same whether we vary $\bar{F}$ or *P*co_2_. This shows that variations in mean molecular weight associated with the compositional variations we consider have a much smaller effect on *P*_res_ than variations in $\bar{T}$. To find the values of *P*co_2_ for a given age (or Solar luminosity), resonant period *P*_res_, and $\bar{T}$, we interpolate between the data points returned by PlaSim.

All the GCM curves are qualitatively similar to those of the one-dimensional models in (*2*); their model c is depicted by the orange dotted line in Fig. 5. Note that the GCM models show much more curvature than the one-dimensional model, a feature that is clearly important and deserves future study.

The geological data in Fig. 3 suggest that *P*_res_ ≈ 19.5 to 20 hours between 2000 and 1000 Ma. Figure 5 shows that this value of *P*_res_ corresponds to a mean global surface temperature of ≈40 to 55°C; all the models shown in Fig. 5 agree on this point. This value of $\bar{T}$ is uncomfortably high, in more than one sense. It is driven by the small error bars on the cyclostratigraphic data point at 1400 Ma, which, as noted above, assumes that *L*_EM_ has remained constant since that epoch.

Our PlaSim GCM simulations, in agreement with earlier work (*51*–*53*), can reach global mean temperatures $\bar{T}\approx 50^{\circ }$C or higher at an age of 1500 Ma, although the Solar luminosity then was ≈88% of what it is today; (LMD-G simulations reach somewhat lower $\bar{T}\approx 40$°C, see fig. S5). The models require *P*co_2_ ≈100 to 200 mbar to reach such high mean temperatures. Recent work does suggest high *P*co_2_ at earlier epochs (*54*, *55*), although the estimates are below *P*co_2_ ≈100 mbar at an age of 1500 Ma.

The GCMs also predict that the mean surface temperature increases with increasing total atmospheric pressure, most plausibly produced by higher N_2_ levels in the ancient atmosphere (*56*). There is evidence, in the form of fossil rain drop impact craters (*57*, *58*) and from fluid inclusions in quartz (*59*, *60*), suggesting that the total atmospheric pressure was not much higher in the past than it is today. These results are in tension with the roughly constant day length seen in Fig. 3, if that constant day length is due to thermal tides and if pressure broadening and the associated opacity increase are at least partially responsible for the higher sound speed.

The atmospheric resonance found by both LMD-G and PlaSim is broad, indicating that a significant fraction, of order 10%, of the energy in the thermal wave is dissipated every day or, in other words, that *Q*_th_ ≈ 10 (see figs. S2 and S4). The origin of this high dissipation rate is unclear. LMD-G and PlaSim use different numerical schemes to model the atmospheric dynamics. We have varied the artificial viscosity of both codes, finding no significant variation in *Q*_th_. We also varied the spatial resolution, finding that *P*_res_ does not change but that *Q*_th_ increases slightly with increasing resolution. Previous analytic estimates suggested that *Q*_th_ was in the range of 20 to 100 (*2*, *61*). The dependence of *Q*_th_ on resolution suggests that the results of both GCMs for this quantity may be artificially low.

Our referee, K. Zahnle, points out that it may also be that the line is broadened by winds or variations in atmospheric properties with longitude and latitude. In the dynamical modeling described below, we treat *Q*_th_ as a free parameter, with a lower limit of 10 and an upper limit of 120.

Changes in atmospheric composition and surface pressure also affect the strength of the pressure perturbation. In our GCM calculations, increasing the total pressure from *P*_s_ = 1 bar to *P*_s_ = 2 bar increases the peak pressure perturbation by a factor of 50%, with negligible change in *Q*_th_ (fig. S2). Changes in the composition (in particular, increasing the amount of CO_2_) can also increase the peak pressure perturbation by a factor of 2.

### Dynamical results

The dynamical equations depend on a large number of parameters, so we resort to Monte Carlo calculations to find the best-fit models (see section S5 and fig. S6), using the emcee package (*62*).

Our parameter set **θ**, not to be confused with the Hough functions Θ, consists of sixteen quantities (listed in table S2). The first two are the initial value of *L*_EM_ and a parameter related to the formation age of the Moon, specifically, the age at which the Lunar semimajor axis reaches 20*R*_⊕_, denoted by τ_☾_. This choice is somewhat arbitrary but was motivated by a desire to have the time between the formation of the Moon and the start of our integrations be less than a hundred million years or so. The next four parameters relate to the properties of the gravitational and thermal tides: the normalization of the ocean model quality factor *Q*_1_, the quality factor *Q*_th_ of the atmospheric resonance, and two parameters associated with the time-dependent atmospheric torque amplitude *A*(*t*), *A*_1_ and *A*_2_. The final parameters are *n*_th_ values of *P*_res_(*t*_th,*i*_), specified at epochs given by *t*_th,*i*_, with *i* running from 1 to *n*_th_; for the full physics model run we present, we use *n*_th_ = 10. The central ages t_th, *i*_ can be found in the note at the bottom of table S2.

**Fig. 6.**
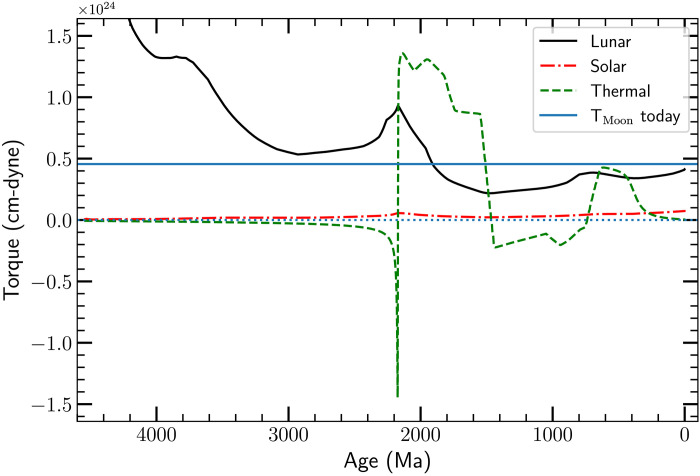
Gravitational and thermal torques as a function of age. The Lunar torque *T*_☾_ (solid black curve), Solar torque *T*_⊙_ (dash-dotted red curve), and atmospheric thermal torque *T*_th_ (dashed green curve) for our best-fit dynamical model. The horizontal solid blue line shows the present day value of the Lunar torque. Note that our convention is that *T*_☾_ and *T*_⊙_ are positive when they remove angular momentum from the spin of Earth, while *T*_th_ is positive when it adds angular momentum to the spin of Earth; this makes it easy to compare the magnitudes of the three torques when the system is in the resonant state. The Earth-Moon system first enters the torque-balanced state around ≈2200 Ma, at which time *T*_th_ becomes positive. The system leaves the balance around ≈1400 Ma. Over this epoch, the lod in the best-fit full model decreases, see Fig. 2. There is a second, brief resonant state between ≈600 and ≈400 Ma. Over this epoch, the lod in the model is constant.

Figure 5 shows the three torques *T*_th_, *T*_☾_, and *T*_⊙_ for our best-fit full model. The Lunar tidal torque (the solid black line in the figure) dominates the dynamics over most of geologic time. For epochs earlier than ≈2300 Ma, the lod is much shorter than twice the atmospheric resonant period *P*_res_ (see Fig. 3 and fig. S7); as a result, *T*_☾_ >> ∣*T*_th_∣, and the thermal tide has little effect on the dynamics. Note that at these early times, both torques act to slow the spin of Earth; following our sign convention, during these epochs, *T*_th_ < 0, as shown in Fig. 6.

However, as the lod increases (due to the Lunar tidal torque), it approaches and eventually equals 2*P*_res_. This occurs at ≈2200 Ma; during the approach to resonance, *T*_th_ increases rapidly in magnitude but remains negative. As the system passes through the resonance, *T*_th_ abruptly changes sign, and, very quickly, *T*_th_ exceeds *T*_☾_. This resonant state lasts until ≈1500 Ma. In this first resonant state, we find *T*_th_/*T*_☾_ ≲ 3. The lod in the model decreases, while the resonance holds (see Fig. 3, black solid line, between 2400 and 1500 Ma). The resonant state is broken around 1500 Ma, after which the lod increases once again. Immediately after the resonance is broken, the resonant period is longer than the lod (2*P*_res_ > lod), so the thermal tidal torque is negative and remains so until ≈700 Ma.

At 700 Ma, there is a second, somewhat shorter resonant or near-resonant epoch, which ends around ≈400 Ma. During this period, the lod in our best-fit model is again fixed, with lod ≈22 hours. The data are rather sparse, but it may be consistent with a second resonant lock.

As described above, we have allowed for the amplitude *A*(*t*) of the thermal torque to vary with epoch. We do so since we are testing the hypothesis that the thermal tide was important in the past and is responsible for the increase in *L*_EM_ we infer from the data in Figs. 2 and 3. Since the present day, thermal torque is far too small to account for the observed increase in *L*_EM_, the torque normalization must have been higher in the past. Indeed, our best-fit model prefers a high normalization (*A*_2_ = 3.51) before 2000 Ma, and a much smaller best-fit normalization *A*_1_ = 0.17 (or median posterior value *A*_1_ = 0.10) at present (see Fig. 4). This reduction in the thermal tide torque over time, coupled with the roughly flat Lunar tidal torque after ≈1800 Ma (Fig. 6) ensures that the resonance state will break. If the reduction in the torque normalization does not break the resonance, some other mechanism is required to do so, e.g., a sharp variation in $\bar{T}$ associated with an ice age (*2*, *63*).

We note that the errors on the data points from (*17*) (the filled green squares in Figs. 1 to 3) are much smaller than the errors associated with any of the other data; the logarithm of the likelihood function in our MCMC calculation is inversely proportional to the error in the data, so the MCMC calculation strongly favors trajectories that pass through those data points. Those data points implicitly assume that *L*_EM_ is constant. As a result, the MCMC calculation favors solutions that conserve *L*_EM_ after 1400 Ma, i.e., solutions for which the thermal torque is small. This is likely why the MCMC best-fit solution breaks the resonance around 1500 Ma.

We regard this extremely low late time thermal torque as nonphysical. In future work, we will relax the assumption of constant *L*_EM_ in analyzing cyclostratigraphic data. Other researchers using cyclostratigraphic techniques might also want to allow for the possibility that *L*_EM_ is not constant.

The Monte Carlo calculation also returns best-fit values and distributions for the other parameters **θ** (see section S6, fig. S6, and table S2) and, in particular, for the resonant period *P*_res_ at various epochs, which we denote by *P*_res,*i*_. We find that the resonant period varies between 23 and 18.5 hours; the best-fit values and 16th and 84th percentile ranges are shown in fig. S7.

**Fig. 7.**
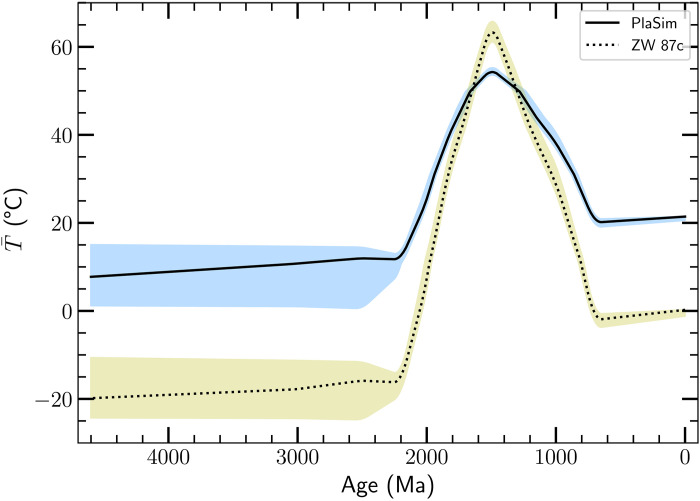
The global mean surface temperature $\bar{T}$ as a function of age. The temperature depicted by the black solid line is inferred from *P*_res_ for our best-fit model, using our GCMs (the solid black curve in Fig. 4), while the dotted curve uses model c from (*2*) (the orange dotted line in Fig. 4). The moderate *T* ≈ 20 or 0^β^C around 0.6 Ga is consistent with the evidence for global glaciation. The high temperatures *T* > 30°C between ≈2 and ≈0.8 Ma are consistent with the lack of large-scale glaciation over that time period (the boring billion). The time average $\bar{T}\approx 40{}^{\circ }\;\mathrm{to}\;45$°C over the boring billion. There is a fairly sharp peak of $\bar{T}\approx 55{}^{\circ }$C at 1500 Ma. We believe that this is the result of the very small error bar assigned to the data in (*17*), combined with the assumption that *L*_EM_ is constant. The shading bracketing the solid and dotted lines shows the range of mean temperatures between the 16th and 84th percentiles of the temperature probability distribution functions (PDFs) for the corresponding model. Note that there is data going back to 3.2 Ga; the weak constraints on $\bar{T}$ for times before 2.8 Ga arise from the very short lod, less than 17.5 hours (see Fig. 2). This is far enough from *P*_res_ for any physically plausible temperature that the thermal tides have little effect on the evolution. As a result, the data cannot constrain $\bar{T}$ at early times, although they can constrain the number of days per month and the lod at early times.

Figure 7 shows the mean surface temperature of Earth corresponding to the resonant period *P*_res_(*t*) in our best-fit dynamical model. The value of $\bar{T}$ returned by the model at early times is consistent with our prior, which is that the climate was temperate during the late Hadean through the Archean (4000 to 2200 Ma).

This contrasts with the result for ages less than ≈2200 Ma. The mean surface temperature of our best-fit dynamical model increases by about 50°C between ≈2200 and ≈1500 Ma. It then drops rapidly to a moderate (≈20°C) and steady value by 600 Ma. As remarked above, temperatures as high as 55^β^C are produced by our GCMs, even for the low Solar flux 1500 Ma ago.

Geochemical data, which also provide estimates of $\bar{T}$, show substantial variation over the past 600 Ma. Our method relies on the integrated thermal torque, so it averages over and thus misses short time (tens of million years) variations.

**Fig. 8.**
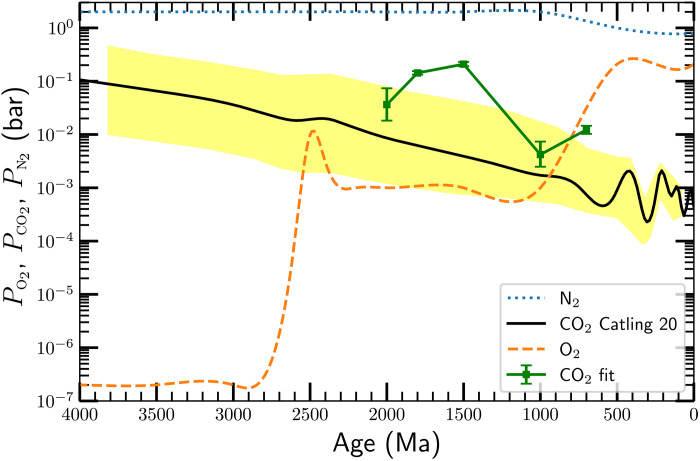
The partial pressures of various atmospheric components over geologic time. The dotted blue line shows our assumed *P*n_2_, which, at early times, is twice the current value, while the dashed orange line shows *P*o_2_, taken from (*54*). The *P*co_2_ levels needed to produce our inferred values of *P*_res_ at 700, 1000, 1500, 1800, and 2000 Ma are shown by green squares with error bars, connect by a sold green line. The solid black line shows *P*co_2_ (yellow shading shows the 95% confidence interval) from (*54*). Our estimates for the CO_2_ levels are at or above the 95% confidence levels reported by those authors, but we note that there are no data between 1.1 and 2.0 Ga reported there, so that the black line is an interpolation rather than a measurement.

We can combine the results in Figs. 5 and 7 to estimate the *P*co_2_ required to reach the values of $\bar{T}$ and hence *P*_res_ at various epochs. Figure 8 shows the result of doing so. It shows the input *P*n_2_ and *P*o_2_ [the latter taken from (*54*)] we adopted (the dotted blue line and the dashed orange line), as well as the values of *P*co_2_ we find at the epochs 700, 1000, 1500, 1800, and 2000 Ma ago (the green squares with error bars, connected by green solid lines).

The figure also shows the estimated *P*co_2_ from (*54*) (the solid black line; yellow shading shows the 95% confidence limits). We infer values similar to those from other sources at 700 to 1000 Ma; at earlier times, our estimates are near or even above (at 1500 Ma) the 95% confidence level shown by the yellow shading. However, we note that those authors quote no measurements between 2000 and 1200 Ma, i.e., the black curve in Fig. 8 is an interpolation rather than a measurement over this time span.

## DISCUSSION

Between 2500 and 2200 Ma, there is good evidence for large-scale glaciations, the Huronian/Makganyene events (*64*, *65*), indicating moderate to low global temperatures and, hence, long *P*_res_. The evidence for glaciers includes diamictites or glacial till and drop stones. Neoproterozoic glaciation events are associated with systematic variations in the isotopic abundance of carbon-13, δC^13^ (*66*); similar variations are seen in some of these early glaciations (*67*), further supporting the interpretation of a moderate or cool global climate. The oldest known glacial deposits, which do not appear to be as extensive as the Huronian events, date back to about 2.8 to 2.9 Ga ago (*68*–*70*). This evidence for glaciation is why we use a prior for the mean *P*_res_ = 22.8 hours at early times, and the strong evidence for Huronian/Makganyene glaciation motivates the small SD σ = 0.1 hours we use for our prior at 2200 Ma (see fig. S8).

The Huronian glaciogenic deposits are overlain by cap carbonates in North America (*64*, *65*, *71*, *72*), India (*73*), and South Africa (*67*). This is similar to the pattern seen (*74*, *75*) in the much younger (neoproterozic) Sturtian and Marinoan glaciations, about 715 and 640 Ma ago. The cap carbonates are believed to form soon after or contemporaneously with deglaciation. The deglaciation itself likely occurs because of the build-up of large amounts of atmospheric CO_2_, resulting in very high mean surface temperatures. Models suggest temperatures as high as $\bar{T}\approx 50{}^{\circ }$C are needed to break out of global scale glacial events, e.g., (*76*).

No large-scale glaciations are known between the Huronian-Makganyene glaciations 2.45 to 2.2 Ga ago and the neoproterozic Sturtian and Marinoan glaciations about 715 and 640 Ma ago. There is evidence of small scale glaciation at ≈1.8 to 1.6 Ga ago (*77*–*79*).

The terms “boring billion” or “barren billion” (*80*, *81*) refer to the period between ≈1800 and ≈800 Ma, which lacks evidence of glaciation and is known to have very stable carbon isotopic ratios (*82*, *83*). The absence of large-scale glaciations motivated the suggestion that the mean surface temperature of Earth was elevated over this period (*84*, *85*). This argument is bolstered by the low number of days per month indicated by the tidal rhythmites before 2000 Ma (Fig. 2) and by the associated short and roughly constant lod, which, given the relation between *P*_res_ and $\bar{T}$, implies a high mean global surface temperature, as shown in Fig. 5.

We associate capture of the Earth-Moon system into torque balance, which occurs at ≈2200 Ma in our best-fit model (Fig. 6), with a rapid mean global temperature rise at about 2200 Ma. Figure 3 shows that before 3000 Ma, the lod ≲ 17.5 hours, shorter than any plausible lod_res_ = 2*P*_res_ for any of our models. By 1850 Ma, the data indicate lod ≈ 19.5 hours. A rapid rise in $\bar{T}$ leads to a rapid drop in *P*_res_; from Fig. 5, we predict that the atmospheric resonant period drops from $P_{\mathrm{res}}(\bar{T}=0)\approx 11.5$ hours to $P_{\mathrm{res}}(\bar{T}=50)\approx 9.5$ hours, resulting in capture into the resonant state.

It is possible that the passage through resonance was simply the result of Lunar tidal torques increasing the lod. Passage through resonance changes the state of the atmosphere, since the time of the local surface pressure maximum abruptly switches from 2:00 a.m. (and p.m.) when lod < 2*P*_res_ to 10:30 a.m. (and p.m.) when lod > 2*P*_res_ (as it is today). This rapid switch is seen in our GCM calculations; it results in a rapid change in the sign of the thermal torque around 2200 Ma seen in Fig. 6. However, this change does not result in a significant change in $\bar{T}$, at least over the duration of our GCM simulations.

We conclude that it is unlikely that the change in the sign of the thermal torque leads to an increase in $\bar{T}$; rather, an increase in $\bar{T}$ leads to a reduction in *P*_res_ at fixed lod and, hence, to a change in the sign of the (near-resonant) thermal torque *T*_th_.

Our results raise the possibility of testing GCMs under significantly different Solar flux and atmospheric composition regimes using Proterozic data, as in Figs. 7 and 8. This is in addition to using Proterozic and or Archean data to constrain the spin history of Earth (Figs. 2 and 3). Known sites for Archean tidal rhythmites include the Keskarrah Formation near Point Lake, Northwest Territories, Canada (*86*, *87*) at an age of 2.6 Ga; the Bell Lake Group in the Slave Craton (*88*), older than 2.8 Ga; and the Randfontein Formation at 2.7 to 3.0 Ga (*89*). As noted above, a completely different technique, cyclostratigraphy (*16*), has recently been used to measure the Lunar semimajor axis and, under the assumption of constant *L*_EM_, the lod at 1375 and 55 Ma (*17*). The assumption that *L*_EM_ is constant should be dropped in future work . It should be possible to use the latter technique on core samples taken at or near the sites where tidal rhythmites are found, providing a check on both methods. The availability of this more firmly established data would, in turn, allow for better constraints on GCMs, potentially increasing their robustness.

Collection of data at the Archean sites listed above will allow for a decisive test of the idea that *L*_EM_ was smaller during that epoch and hence of the idea that the thermal torque was important in the distant past. It is also worth emphasizing that the estimate of the mean surface temperature shown in Fig. 7 provides an independent check on geochemical temperature estimates.

If we integrate the initial conditions from our best-fit thermal tide model but neglect the thermal tide, we find a lod of about 65 hours at the current epoch. The period of the normal mode of Earth’s atmosphere is set primarily by *R*_⊕_ and the mean surface temperature, which combine to give *P*_res_ ≈ 9 to 11.5 hours. The data shown in Fig. 3 indicate that the lod was roughly constant over the boring billion, consistent with capture into a state in which *T*_☾_ ≈ *T*_th_. The long duration and relatively recent occurrence of this resonant state may be responsible for the fact that the day is currently 24 hours long.

## MATERIALS AND METHODS

### Resonant lod

We determined the lod_res_ (i.e., when the lod equals twice the period of the westward going *s* = 2 symmetric wave) from our GCMs as follows. We start by specifying a set $\text{Λ}=(\bar{F},{\bar{P}}_{s},Z)$ and lod then run the GCM for several to tens of simulated years, until the model reaches a mean thermal equilibrium. We then calculate the 2-year time-average semidiurnal component $P_{2}^{2}$ of the pressure variation (equivalent to the quantity approximated in eq. S25). We repeat the calculation at a number of different values of lod, holding Λ fixed. The resonant period *P*_res_ for that Λ can then be inferred from a plot of the semidiurnal pressure perturbation (fig. S2) or the torque (fig. S4) as a function of lod.

To determine the frequency of westward going *s* = 2 symmetric wave, we run a single model long enough to reach thermal equilibrium then produce a dispersion relation, i.e., the power spectrum as a function of zonal wave number and frequency (*s*, ω). To do so, we perform a Fourier transform of the surface pressure perturbation *P*_s_(θ, ϕ, *t*), a function of colatitude, east longitude, and time, both spatially and temporally. The normal or free oscillations show up as peaks in the power at discreet values of *s* and ω, with the westward traveling mode at *s* = 2, and a frequency slightly higher than 2.0 cycles/day; we find ω = 2.097 cycles/day or a period of 11.44 hours. The torque normalization *A*(Λ) can be read off a plot of either the thermal pressure perturbation $P_{2}^{2}$ or the tidal torque *T*_th_ versus lod.

### Integrating the equations of motion

We use the scipy integrator scipy.integrate.odeint to integrate the equations of motion, Eqs. 6 to 8, with parameters rtol = atol = 3 × 10^−10^.

Note that *L*_⊕_ >> *L*_EM_ and that the fractional variation ∣δ*L*_⊕_∣/*L*_⊕_ ≈ 3 × 10^−8^, so the effects of such variations on the evolution of *S*_⊕_ and *L*_☾_ could be neglected. However, we have kept *L*_⊕_ as a dynamical variable so that we can check how well the total angular momentum *L*_⊕_ + *L*_☾_ + *S*_⊕_ is conserved by our integration. We find that the fractional error in the total angular momentum is of order 3 × 10^−15^, much less than ∣δ*L*_EM_/*L*_⊕_∣, indicating that the integration is reliable.

## Supplementary Materials

### This PDF file includes:

Sections S1 to S6 Figs. S1 to S7 Tables S1 and S2 References
