# Supplementary material for: Why the day is 24 hours long: The history of Earth’s atmospheric thermal tide, composition, and mean temperature
Source: Sci Adv. 2023 Jul 5;9(27):eadd2499. doi: 10.1126/sciadv.add2499 (PMC10321735; doi:10.1126/sciadv.add2499)
Supplement: Supplementary file 1 — Sections S1 to S6 Figs. S1 to S7 Tables S1 and S2 References [file sciadv.add2499_sm.pdf]

Science Advances
AAAS

Supplementary Materials for

Why the day is 24 hours long: The history of Earth's atmospheric thermal tide, composition, and mean temperature

Hanbo Wu *et al.*

Corresponding author: Norman Murray, murray@cita.utoronto.ca

Sci. Adv. **9**, eadd2499 (2023)
DOI: 10.1126/sciadv.add2499

This PDF file includes:

Sections S1 to S6
Figs. S1 to S7
Tables S1 and S2
References

Supplementary Material

S1 Simplified Gravitational Tide Model

As stated in the main text, we use a simple tidal model which neglects the obliquity of Earth as well as the eccentricity and inclination of the Moon's orbit, in which the the Lunar tidal torque is (*35, 36*)

$$T_{☾} = \frac{3}{2}\frac{Gm_{☾}^2}{a_{☾}}\left(\frac{R_\oplus}{a_{☾}}\right)^5 \frac{k_2}{Q_1 Q(\omega_{☾}^t)}. \qquad \textbf{(S1)}$$

The tidal dissipation on Earth occurs primarily in shallow seas, with an approximately 30% contribution from the deep ocean (*91*). It is known that the shape and even the number of ocean basins have changed over time. Reconstructions of the past disposition of the continents and ocean basins have been made. These have then been used to calculate the ocean tides and dissipation, e.g., (*3*) who did so over the last 250 Myr.

However, we require a calculation of the tides going back more than four billion years, so we settle for a much simpler tidal model (*6, 92*). The model consists of a single hemispheric ocean (similar to the Pacific ocean) with a constant depth. Laplace's tidal equations, with a linear friction term, are solved, with the boundary condition that there is no flow across the coastline. The tides are driven at both diurnal and simi-diurnal periods. The model exhibits resonances in the tidal response. The power dissipated in the tide varies strongly with the period of the forcing, with a general decrease in dissipated power with increasing tidal frequency (or decreasing tidal period). This result, expressed in terms of the effective tidal quality factor $Q(\omega)$, is depicted in Fig. S1, which shows that $Q(\omega)$, which is inversely proportional to dissipated power, tends to increase with increasing tidal frequency ω although $Q(\omega)$ is not monotonic; the features are associated with resonances, and are very model dependent.

The Solar tidal torque, $T_\odot$, is usually taken to be proportional to T_L, a practice we will follow: $T_\mathrm{L} + T_\odot = T_\mathrm{L}(1+\beta)$, where

$$\beta = \frac{1}{4.7}\frac{Q(\omega_{☾})}{Q(\omega_\odot)}\left(\frac{a_{☾}}{a_{☾,0}}\right)^6 \qquad \textbf{(S2)}$$

where $a_{☾,0}$ is the present day value of the Lunar semimajor axis, while $\omega_\odot^t \equiv 2(\Omega_\oplus - n_\oplus)$, with $n_\oplus = \sqrt{G(M_\odot + M_\oplus)/a_\oplus^3}$ being the Earth's mean motion. The factor $1/4.7$ is the present day ratio of $T_\odot/T_{☾}$; since $a_{☾}/a_{☾,0}$ was less than one in the past, the Solar tide was relatively less important then.

The Earth also exhibits solid body tides. We adopt a solid body tidal $Q_s = 250$, and use the minimum of Q_s and $Q(\omega)$. This affects only the very early evolution of the system.

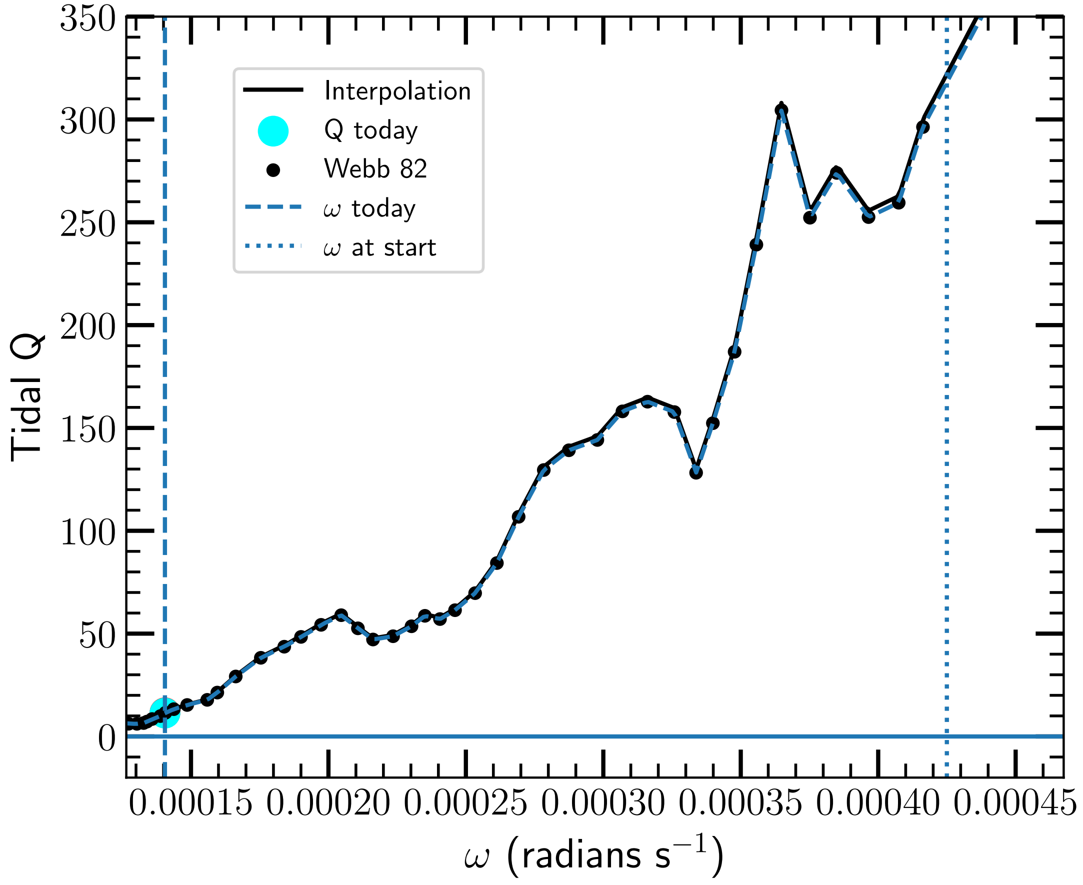

Fig. S1: The tidal dissipation factor $Q(\omega)$, from the ocean model of reference (*6*). The tidal Q increases rapidly with increasing frequency, i.e., with decreasing length of day. This effect tends to decrease the torque and power dissipated by tides in the past, when the length of day was shorter than 24 hours. This tendency is opposed by the higher tides produced by the Moon in the past, when it was closer to Earth. The frequency corresponding to 24 hours is indicated by the vertical dashed line. The vertical dotted line shows the tidal frequency corresponding to the situation at the start of our integration, for the best fit full thermal and gravitational tide model. Recall that for the Lunar tide, $\omega = \omega^t = 2(\Omega_\oplus - n_☾)$.

Table S1: Geophysical Parameter Values

Quantity	Symbol	Value	Unit
Gravitational constant	G	6.67428×10^{-8}	$\mathrm{g^{-1}\ cm^3\ s^{-2}}$
Solar mass	$M_\odot$	1.98842×10^{33}	g
Earth Mass	$M_\oplus$	5.97219×10^{27}	g
Lunar Mass	$M_☾$	7.34581×10^{25}	g
Earth semimajor axis	$a_\oplus$	1.49597×10^{13}	cm
Earth mean motion	$n_\oplus$	1.990987×10^{-7}	rad $\mathrm{s^{-1}}$
Earth radius	$R_\oplus$	6.37101×10^{8}	cm
Lunar semimajor axis	$a_☾$	3.84402×10^{10}	cm
Lunar mean motion	$n_☾$	2.6617×10^{-6}	rad $\mathrm{s^{-1}}$
Earth Polar moment	C	$0.3307007 M_\oplus R_\oplus^2$	g $\mathrm{cm^2}$
Earth Love number	$k_{2,\oplus}$	0.298	—
Solar day length	$\tilde{P}_\oplus$	86400	s
Sidereal day length	$P_\oplus$	86164	s
Thermal Torque today	$T_{\mathrm{th}}(0)$	4.14×10^{22}	dyne cm

Note–Values from (*45*), except for G and $M_\oplus$, which are from the IAU.

S2 Simplified Thermal Tide Model

We present a derivation of a simple one-dimensional thermal tide model, which captures the relevant resonant wave behavior in a one dimensional model along the Earth's equator. The model neglects the rotation of Earth (the Coriolis and centrifugal forces), and the vertical variation of temperature and deposition of the rate of energy deposition ($\dot{q}$). We start with first law of thermodynamics,

$$de = Tds - Pd(1/\rho), \tag{S3}$$

where e is the internal energy per gram of air, s is the entropy per gram, T is the temperature, P the pressure, and ρ the mass density. Air is adequately described as an ideal gas with five degrees of freedom, so that the specific heat at constant volume

$$c_v \equiv \left(\frac{dq}{dT}\right)_V = \left(\frac{de}{dT}\right)_V = \frac{5}{2}\frac{k_b}{\mu}, \tag{S4}$$

where $dq = Tds$ is the change in heat (thermal energy) per gram, $\mu = 28.96 m_p$ is the molecular weight of air, (with $m_p = 1.67 \times 10^{-24}\,\mathrm{g}$ the proton mass), and $k_b = 1.38 \times 10^{-16}\,\mathrm{erg\,K^{-1}}$ is Boltzman's constant.

The equation of state for an ideal gas is

$$P = \rho k_b T/\mu. \tag{S5}$$

For later reference $\rho \approx 1.2 \times 10^{-3}\,\mathrm{g\,cm^{-3}}$ and $P \approx 1.013 \times 10^{6}\,\mathrm{dynes\,cm^{-2}}$ (*93*) at the surface of Earth.

It follows that $e = c_V T$.

The magnitude of the thermal tide is easily measured using the surface pressure, so we will use pressure as the dependent variable. Later we will introduce the momentum and mass conservation equations, which depend on P and ρ, and not on T, so we use the equation of state to change variables from T to ρ:

$$c_v dT = \frac{c_v \mu}{k_b} d\left(\frac{P}{\rho}\right) = \frac{c_v \mu}{k_b}\frac{P}{\rho}\left[\frac{dP}{P} - \frac{d\rho}{\rho}\right]. \tag{S6}$$

Using this in equation (**S3**), we obtain

$$\frac{c_v \mu}{k_b}\frac{dP}{\rho} = dq + \left(c_v + \frac{k_b}{\mu}\right)\frac{\mu}{k_b}\frac{P}{\rho^2} d\rho, \tag{S7}$$

or

$$\frac{1}{\rho} dP = \frac{k_b}{c_v \mu} dq + \frac{c_p}{c_v}\frac{P}{\rho^2} d\rho, \tag{S8}$$

where we have used the result (following from the ideal gas law) that the specific heat at constant pressure $c_p \equiv (dq/dT)_P = c_v + k_b/\mu$.

The energy equation follows:

$$\frac{dP}{dt} - \gamma \frac{P}{\rho}\frac{d\rho}{dt} = \frac{k_b}{c_v \mu}\rho\, \dot{q}, \tag{S9}$$

where $\gamma \equiv c_p/c_v$ and $\dot{q} \equiv dq/dt$. For dry air at 20 C, $\gamma = 1.4$, consistent with our choice of $c_v = (5/2)(k_b/\mu)$.

The continuity and momentum equations are

$$\frac{\partial \rho}{\partial t} + \nabla \cdot \rho \mathbf{u} = 0 \tag{S10}$$

$$\frac{\partial \mathbf{u}}{\partial t} + \mathbf{u} \cdot \nabla \mathbf{u} + \frac{1}{\rho}\nabla P - \mathbf{g} = -\Gamma \mathbf{u}, \tag{S11}$$

$$\tag{S12}$$

where $\mathbf{u}$ is the fluid velocity. We have introduced a term $-\Gamma \mathbf{u}$, representing drag, into the momentum equation. For future use, we note the relation between the drag coefficient Γ and the quality factor of the atmospheric resonant cavity $Q_{\rm th}$,

$$Q_{\rm th} \equiv \frac{\omega_{\rm th}}{\Gamma}, \tag{S13}$$

where $\omega_{\rm th}$ is the resonant frequency of the relevant atmospheric mode (see below).

Noting that

$$\frac{d\rho}{dt} = \frac{\partial \rho}{\partial t} + \mathbf{u} \cdot \nabla \rho, \tag{S14}$$

we can use the continuity equation in the energy equation

$$\frac{dP}{dt} + \gamma P\, \nabla \cdot \mathbf{u} = \frac{k_b}{c_V \mu}\, \rho\, \dot{q}. \tag{S15}$$

At this point we will make a number of assumptions to simplify the problem. First, we note that the observed pressure fluctuations have an amplitude of about 1 millimeter of mercury, or one millibar, i.e., about one part in a thousand. Thus we write

$$P = P_0 + P_1 \tag{S16}$$

with $P_1 << P_0$, and similarly for ρ. We also assume that the background state consists of the atmosphere at rest, so that $\mathbf{u} = \mathbf{u}_1$. We treat $\dot{q}$ as a first order quantity as well. We keep terms only up to first order in P_1, ρ_1 and u_1.

To lowest order, the momentum equation becomes

$$\nabla P_0 = -\rho_0 \mathbf{g}. \tag{S17}$$

We next use the fact that the vertical extent of the atmosphere is much smaller than the radius of Earth to make the assumption that the atmosphere is plane parallel, with gravity acting in the z

direction, and $\mathbf{u} = (u, v, w)$ in Cartesian coordinates. The x-axis is taken parallel to the Earth's equator, and runs from $-\pi R_\odot$ to $\pi R_\odot$, and the atmosphere is invariant in the y direction, i.e., the y-component v of $\mathbf{u}$ is zero. We remind the reader that we have also neglected the rotation of Earth, ignoring the associated Coriolis and centrifugal accelerations.

Finally, we anticipate that we are looking for a Lamb wave, so that vertical velocity $w = 0$. This precludes an accurate solution for the vertical structure of the mode, with the result that we give up some accuracy in the estimate of the resonant period. This is not as bad as it might seem since our GCM results, when compared with more accurate analytic solutions, show that the latter also do poorly in this regard. The momentum and energy equations become

$$\frac{\partial P}{\partial t} + \gamma P_0 \frac{\partial u}{\partial x} = \frac{k_b}{c_V \mu} \rho_0 \, \dot{q} \qquad \textbf{(S18)}$$

$$\frac{\partial u}{\partial t} + \frac{1}{\rho_0} \frac{\partial P}{\partial x} = -\Gamma u, \qquad \textbf{(S19)}$$

where we have dropped the subscript 1 from all the perturbed quantities.

Taking the partial time derivative of the first equation, then using the second equation to eliminate u, we find

$$\frac{\partial^2 P}{\partial t^2} + \Gamma \frac{\partial P}{\partial t} - c_s^2 \frac{\partial^2 P}{\partial x^2} = \frac{k_b}{\mu c_V} \rho_0 \left[\Gamma \dot{q} + \frac{\partial}{\partial t} \dot{q} \right]. \qquad \textbf{(S20)}$$

The sound speed $c_s \equiv \sqrt{\gamma P_0 / \rho_0} \approx 3.4 \times 10^4 \mathrm{cm\,s^{-1}}$.

To estimate the heating rate per gram of air ($\dot{q}$), we first relate it to the mean heating rate per square centimeter $\bar{F}$, which is the quantity that is usually reported,

$$\dot{q} = \frac{\bar{F}}{\int_0^\infty \rho dz} = \frac{g \bar{F}}{P_0}, \qquad \textbf{(S21)}$$

where we used eqn. (**S17**).

Note that we are interested in the flux absorbed by the atmosphere, not the flux at the top of the atmosphere; the latter is $\bar{S} = 340\,\mathrm{W\,m^{-2}}$ averaged over the surface of the Earth (corresponding to the usual solar constant $S = 1,360\,\mathrm{W\,m^{-2}}$. The mean absorbed flux is estimated by (*94*) to be $75 + 24 + 88 = 187\,\mathrm{W\,m^{-2}}$; the first term corresponds to direct absorption of sunlight, the second to sensible heating from the ground, and the third to latent heating (related to water evaporation). In cgs units the total $\bar{F} = 1.87 \times 10^5\,\mathrm{erg\,cm^{-2}\,s^{-1}}$. Similar values for each term are given in (*95*) ($78 + 17 + 80 = 175\,\mathrm{W\,m^{-2}}$) and in (*96*) ($79 + 20 + 84 = 183\,\mathrm{W\,m^{-2}}$).

The heating due to direct absorption of sunlight in the atmosphere is zero from sunset to sunrise. During the day the direct absorption term is proportional to $\cos(\tilde{\Omega}_\oplus t)$, where $\tilde{\Omega}_\oplus = \Omega_\oplus - n_\oplus = 2\pi/\tilde{P}_\oplus$ and $\tilde{P}_\oplus$ is the length of a Solar day, currently $24\,\mathrm{hr}$ or 86400 seconds. We take the zero of time to be at noon. For simplicity, in this section we will assume that the other two fluxes have the same temporal structure, a rather crude approximation. This assumption is relaxed in our GCM modeling.

Using the notation of reference (*44*), we expand the pressure and the heating rate in Fourier components, and pick out the component that produces the leading term in the torque associated with the thermal tide, namely the semidiurnal (or 12hr period term, currently)

$$P_2 = P_2^2 \exp^{i(2\tilde{\Omega}_\oplus t + 2kx)} \quad \textbf{(S22)}$$

$$\dot{q} = \dot{q}_2^2 \exp^{i(2\tilde{\Omega}_\oplus t + 2kx)} \quad \textbf{(S23)}$$

with $k = 2\pi/(2\pi R_\oplus)$; note that this is half the wave number associated with the semidiurnal tide, i.e., k corresponds to a wavelength equal to the circumference of Earth, while the wavelength associated with the semidiurnal tide is $\pi R_\oplus$, half the circumference; the factor of 2 in the exponents in eqns. **S22** and **S23** accounts for the difference. Inserting these relations into eqn. (**S20**), some algebra yields

$$P_2^2 = \frac{2}{3\pi}\frac{k_b}{\mu c_V}\frac{\rho_0 g}{P_0} 4\bar{F} \frac{\left[\Gamma + 2i\tilde{\Omega}_\oplus\right]\left[4(\omega_{\rm res}^2 - \tilde{\Omega}_\oplus^2) - 2i\tilde{\Omega}_\oplus\Gamma\right]}{16(\omega_{\rm res}^2 - \tilde{\Omega}_\oplus^2)^2 + 4\tilde{\Omega}_\oplus^2\Gamma^2}, \quad \textbf{(S24)}$$

with the factor $2/(3\pi)$ coming from the Fourier transform of the time-dependent heating. We have defined $\omega_{\rm res} \equiv c_s k = c_s/(R_\oplus)$; the frequency in the atmosphere is twice this, $\omega = c_s/(R_\oplus/2) = 2c_s/R_\oplus$, corresponding to a resonant period about $P_{\rm res} = 16.4$ hours for our over-simplified model, compared to $P_{\rm res} = 11.4$ or $P_{\rm res} = 11.5$ hr for PlaSim and LMD respectively.

The perturbed pressure is then

$$\begin{aligned} Re\{P_2\} = {} & \frac{2}{3\pi}\frac{k_b}{\mu c_V}\frac{\rho_0 g}{P_0} 4\bar{F} \\ & \times \left\{ \frac{\Gamma\omega_{\rm res}^2 \cos(2\tilde{\Omega}_\oplus t + 2kx)}{4(\tilde{\Omega}_\oplus^2 - \omega_{\rm res}^2)^2 + \tilde{\Omega}_\oplus^2\Gamma^2} \right. \\ & \left. + \frac{[2\tilde{\Omega}_\oplus(\tilde{\Omega}_\oplus^2 - \omega_{\rm res}^2) + \tilde{\Omega}_\oplus\Gamma^2/2]\sin(2\tilde{\Omega}_\oplus t + 2kx)}{4(\tilde{\Omega}_\oplus^2 - \omega_{\rm res}^2)^2 + \tilde{\Omega}_\oplus^2\Gamma^2} \right\}. \end{aligned} \quad \textbf{(S25)}$$

In the main text, and below, we follow the general practice in the thermal tide literature and use the quality factor of the resonant cavity (the Earth's atmosphere) $Q_{\rm th} = \omega_{\rm res}/\Gamma$, as noted above.

Using $P_{\rm res} = 11.4$ h for the present day Earth, and taking $Q_{\rm th} = 30$, eqn. (**S25**) predicts a pressure perturbation of

$$\delta P_s \approx 1170\,{\rm dyne\,cm^{-2}} \approx 1.2\,{\rm millibar}, \quad \textbf{(S26)}$$

within a factor of two of the observed value at Earth's equator, $\delta P_s \approx 0.8$ millibar (*39, 40*).

Fig. S2 shows the quadrupolar pressure perturbation calculated by PlaSim (the dots, see section **S4** below) as well as a fit of the form given by eqn. (**S25**). We have adjusted the normalization of the fit to minimize the χ^2 between the fit and the numerical results.

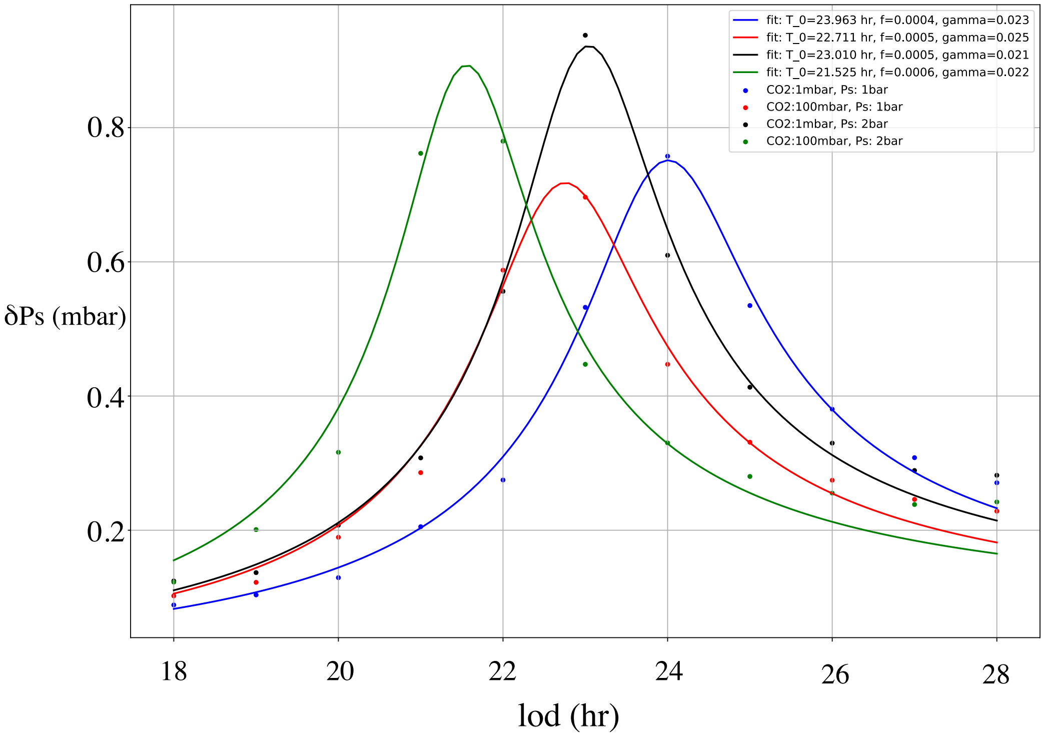

Fig. S2: The amplitude of the quadrupolar pressure perturbation δP_s due to solar heating as a function of the length of day, as calculated by PlaSim (colored points). Each point corresponds to a single simulation, with the length of day $P_\oplus$ given by the value on the x-axis. The solid lines are fits using eqn. (**S25**). The maximum in δP_s marks the value of $P_{\rm res}$, twice the atmospheric resonant period. The blue and red points and curves correspond to a 1 bar atmosphere, with $P_{CO_2} = 1$ mb and 100 mb respectively, while the black and green points and curves correspond to a 2 bar atmosphere and the same P_{CO_2} values. Consider the blue and black curves, which both have $P_{CO_2} = 1$ mbar. When P_s is doubled, the peak in δP_s moves to shorter period, from $P_{\rm res} \approx 24$ hr to $P_{\rm res} \approx 23$ hr. This is the result of two effects; first, the mean surface temperature increases with increasing P_s, and second, the mean molecular weight $\mu(Z)$ decreases; both changes lead to an increase in the sound speed, and hence a lower $P_{\rm res}$. The same two effects explain the decrease in $P_{\rm res}$ between the red and green curves. The height of the peak, corresponding to an increase in the normalization N of the thermal torque, increases with increasing P_s at fixed P_{CO_2} (blue to black, and red to green).

The relation between the perturbed pressure and the torque is (*44*)

$$T_{\rm th} = \sqrt{\frac{24\pi}{5}} \frac{M_\odot}{M_\oplus} R_\oplus^3 \left(\frac{R_\oplus}{a_\oplus}\right)^3 |P_2^2| \sin\delta_{\rm th}, \qquad \textbf{(S27)}$$

where $\delta_{\rm th}$ is the phase where the pressure maximum occurs; for the present-day Earth, this corresponds to a local Solar time of $\approx 10:30$ am and pm. Using this expression, we find

$$T_{\rm th} \approx \sqrt{\frac{24\pi}{5}} \frac{GM_\odot R_\oplus}{g} \left(\frac{R_\oplus}{a_\oplus}\right)^3 \frac{2}{3\pi} \frac{k_b}{\mu c_V} \frac{\rho_0 g}{P_0} 2\bar{F} \cdot f(\tilde{\Omega}_\oplus, \omega_{\rm res}, Q_{\rm th}), \qquad \textbf{(S28)}$$

where

$$f(\tilde{\Omega}_\oplus, \omega_{\rm res}, Q_{\rm th}) \equiv \frac{4\tilde{\Omega}_\oplus(\tilde{\Omega}_\oplus^2 - \omega_{\rm res}^2) + \tilde{\Omega}_\oplus \omega_{\rm res}^2/Q_{\rm th}^2}{4(\tilde{\Omega}_\oplus^2 - \omega_{\rm res}^2)^2 + \tilde{\Omega}_\oplus^2 \omega_{\rm res}^2/Q_{\rm th}^2}, \qquad \textbf{(S29)}$$

which represents the response of a damped driven harmonic oscillator. The f function has units of inverse frequency, or time.

The torque is maximized when $\tilde{\Omega}_\oplus \approx \omega_{\rm res} = c_s k$, or when the rotation period is $\tilde{P}_\oplus = 1/2 \cdot 2\pi R_\oplus / c_s$. Using the present day surface sound speed $c_s = 3.4 \times 10^4 \ {\rm cm\,s^{-1}}$, the resonant rotation period is about ${\rm lod_{res}} \approx 33$ hours, compared to a period just under 23 hours found by our GCM models, so our crude approximations provide estimates good to about 40%, similar to the error in the estimate for δP_s found above.

There are a number of factors in eqn. (**S28**) that can be expected to change significantly over geologic time, two in the prefactor, $P_0/\rho_0 g \equiv H(\mu, T)$ and $\bar{F}(L_\odot(t), Z, P_0))$, and all three arguments of the frequency function, specifically, $\omega_{\rm res}(\mu, T, \tilde{\Omega}_\oplus)$, $Q_{\rm th}(Z, P_0)$, and $\tilde{\Omega}_\oplus(t)$. Recall that we employ Z to denote the variable composition of the atmosphere, and note that $\mu = \mu(Z)$.

It is unclear what sets $Q_{\rm th}$, but candidates include radiative damping, ion damping at altitude, and surface friction (*61*). The GCMs that we employ both apply simple boundary conditions at the top of the model atmosphere, which may also introduce an effective dissipation, e.g., by artificially scattering the traveling sound wave into gravity waves. We will treat $Q_{\rm th}$ as a free parameter, but assume that it does not vary with epoch.

The resonant period $P_{\rm res}$, or equivalently, $\omega_{\rm res}$ depends on the sound speed, which almost certainly varied with epoch, so we treat $P_{\rm res}$ as a time-dependent free parameter when fitting the model to data.

The Solar luminosity $L_\odot$ changes by some 30% over the age of Earth. Following (*48*) we use the approximation

$$L(t) = \frac{L_\odot}{1 + \frac{2}{5}(1 - t/t_\odot)} \qquad \textbf{(S30)}$$

with $t_\odot = 4,560$ My the age of the sun, and $0 \le t \le t_\odot$; the sun formed at $t = 0$, while $t = 4,560$ My corresponds to the present epoch.

Similarly, the composition and the mean surface pressure $\bar{P}_s$ (or mass) of the atmosphere alter the opacity, and both are believed to change with epoch. All three affect $\bar{F}$. For example, simulations using both LMD-G and PlaSim show that increasing the amount of N_2 while keeping the other components fixed increases the surface pressure perturbation (see below). Increasing both P_{CO_2} and P_{N_2} increases the pressure perturbation even more. Increasing the Solar luminosity does as well.

We will treat $\bar{F}/H$ as a time-dependent free parameter in our fits to data. We do so by introducing a dimensionless normalization factor $A(t)$ to describe the torque $T_{\rm th}$:

$$T_{\rm th} = A(t) T_{\rm th}(0) \frac{f(\tilde{\Omega}_\oplus, \omega_{\rm res}, Q_{\rm th})}{f(\tilde{\Omega}_\oplus(0), \omega_{\rm res}(0), Q_{\rm th}(0))}, \qquad \textbf{(S31)}$$

where we use $T_{\rm th}(0) = 4.14 \times 10^{22}\,\mathrm{dyne\ cm}$ for the value of the present day thermal torque. $A(t)$ can change on geologic time scales, and may vary by factors of several.

Fig. S3 shows this torque with $A(t) = A(0) = 1$ and $P_{\rm res} = 22.8$ hr (the solid line) and for $A = 2$, $P_{\rm res} = 19$ hr (the dot-dash line); in both cases we set $Q_{\rm th} = 100$.

We employ a simple piece-wise linear model for $A(t)$:

$$A(t) = \begin{cases} A_1 + (A_2 - A_1)(t/t_0) & t < t_0 \\ A_2 & t \geq t_0. \end{cases} \qquad \textbf{(S32)}$$

This introduces three parameters, two that we vary as part of the Monte Carlo calculation (A_1 and A_2), and one that we consider fixed, the age at which the normalization begins to decrease, denoted t_0. Note that t runs from $4,560$ Ma to the present (0). We have tried various values $1,000\,\mathrm{Ma} < t_0 < 2,500$ Ma, so that $A(t) = A_2$ in the Hadean and Archean. The results do not depend strongly on the exact value, so we set $t_0 = 2,000$ Ma. The dynamical model then prefers $A_1 < A_2$, i.e., the thermal torque was larger in the past than at present, by a factor of several.

In Fig. S4 we compare the torque from eqn. (**S28**) against that calculated by PlaSim for present day values of $\mathbf{\Lambda} \equiv (\bar{F}, Z, \bar{P}_s)$. The fit from eqn. (**S28**) is fairly good away from resonance, and for day lengths $\tilde{P}_\oplus > P_{\rm res}$. The peak torque for $\tilde{P}_\oplus < P_{\rm res}$ (where the torque is negative) is overestimated by eqn. (**S28**) by about 40% compared to the result from PlaSim (results from LMD-G are similar to those of PlaSim).

We use eqns. **S28** and **S29** in our dynamical model rather than the GCM estimate of $T_{\rm th}$ for two reasons. The first is practical; we need to find the torque for hundreds of thousands of dynamical models, so running a GCM is not feasible. The second reason is related to the value of $Q_{\rm th}$. The values predicted by the GCMs are around $Q_{\rm th} \approx 10$, while the analytic predictions range from 20 to 100. The geologic data appear to favor larger values.

It is worth stressing that the GCM result shown in Fig. S4 demonstrates that when $P_\oplus < P_{\rm res}$, the thermal torque tends to increase the length of day, but when $P_\oplus > P_{\rm res}$ (as it is today) the thermal torque tends to decrease the length of day. This is in agreement with the linear theory.

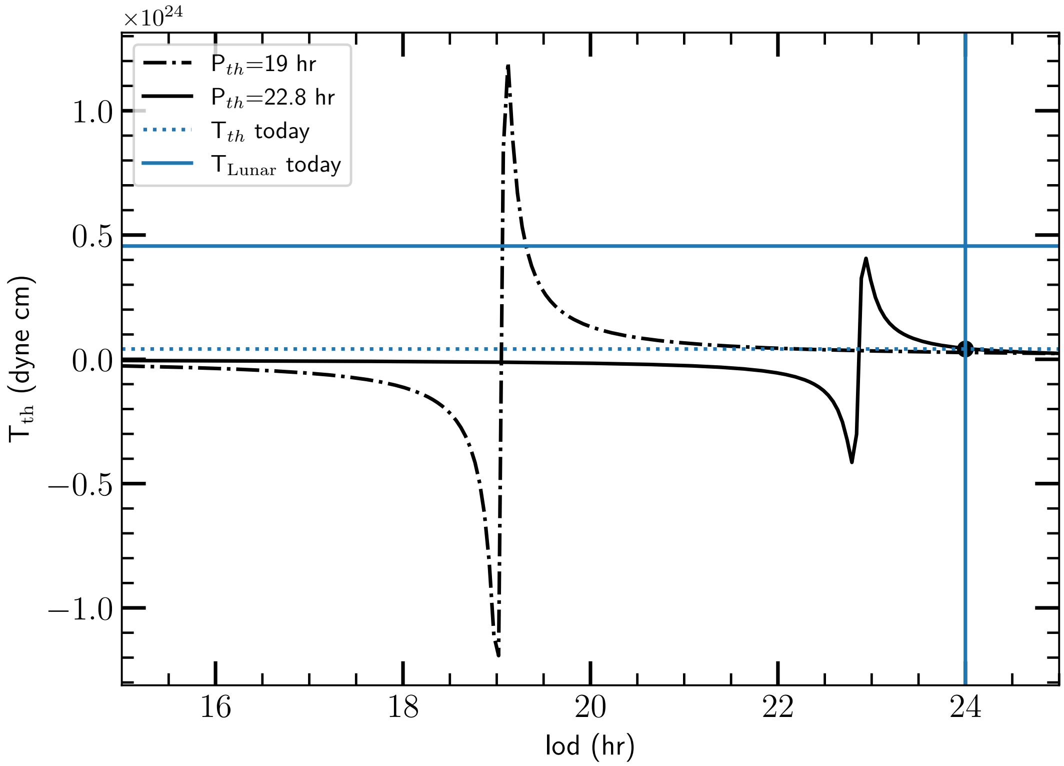

Fig. **S**3: The normalized torque (eqn. **S31**) as a function of the Earth's spin period or LOD. The solid line shows the torque for $N = 1$, $P_{\rm res} = 22.8$ hours, and $Q_{\rm th} = 100$, the first two values corresponding to present-day values, the third suggested by fitting our dynamical model to the geologic data. The dot-dash line shows the torque for values similar to those in the Proterozoic, $N = 3.6$, $P_{\rm res} = 19$ hours, and $Q_{\rm th} = 100$. The horizontal dotted line shows the present day thermal torque $T_{\rm th}(0) = 4.14 \times 10^{22}\,{\rm dyne\,cm}$ ignoring any ocean response, while the horizontal solid line shows the present day value of the Lunar tidal torque, $T_{☾}(0) = 4.46 \times 10^{23}\,{\rm dyne\,cm}$.

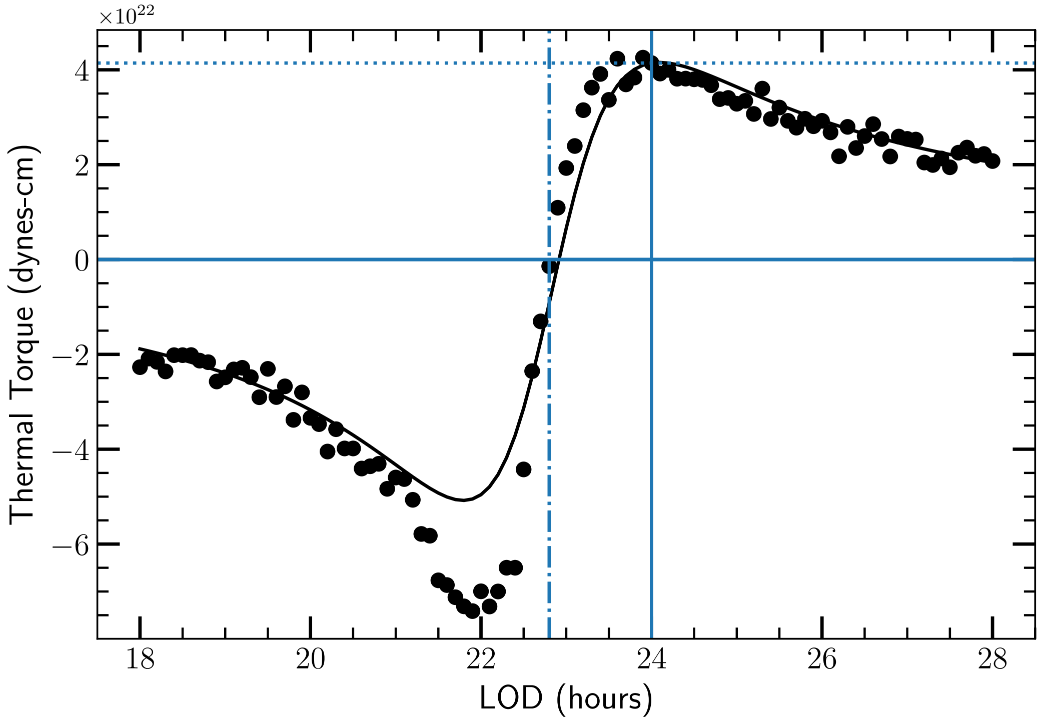

Fig. S4: The atmospheric thermal torque as a function of the length of day given by PlaSim (dots). The solid curve shows the prediction of eqns. **S28** and **S29**. Both results have been normalized to the estimated present day value at $\tilde{P}_{\oplus} = 24\,hr$, shown by the dotted horizontal line. The dot-dash blue vertical line shows the value $P_{\rm res} = 22.8$ inferred for this set of PlaSim simulations.

S2.1 Reduced torque due to isostatic adjustment in oceans

Equation (**S28**) gives the torque produced by the Sun's gravity on the mass quadruple of the atmospheric tide. However, if the atmospheric bulge lies over the ocean, the ocean will respond to the excess pressure (over the mean atmospheric pressure); the excess pressure in the body of the ocean will tend to produce horizontal motions away from the maximum pressure, much like a barometer reacts to increases in atmospheric pressure. In fact, the tendency of the ocean to act like an inverse barometer in response to pressure loading is well known, see, e.g., chapters 6 and 7 in (*37*). Reference (*90*) provides a nice review. As an extreme limit, eqn. (4.13) in (*97*) shows that an atmospheric mass (and hence pressure) perturbation over the ocean can be exactly compensated for by an opposite perturbation in the ocean. Thus the torque from the thermal tide is reduced by about 75% from that calculated here, see his eqn. (4.16).

Recently the effect of the semidiurnal thermal tide on the pressure at the bottom of the ocean has been directly detected using both satellite altimetry (*98*) and satellite gravimetry (*99*). The first paper compares satellite measurements of the height of the ocean to direct measurements of the pressure at 39 barometric pressure recorders on the open ocean floor in the tropics (since the thermal tide is small outside the tropics). The effects of both the diurnal and semidiurnal tides were detected. The results show that at 12 hours and over the scale of the tide, the ocean acts as an imperfect inverse barometer, with an ocean floor pressure reduction of about 70% of that of a perfect inverse barometer. The second paper uses gravimetry from the GRACE and GRACE-FO missions, combined with tide gauge and the ocean floor data just discussed, and shows that accounting for the diurnal and semidiurnal atmospheric tides (as well as higher frequency tides) improves the fits. Both papers are able to separate out the effects of the thermal tides from the gravitational tides.

For an example of the opposite limit, meaning a very small inverse barometer effect, consider a planet with oceans confined (by continents) to regions between meridional lines running from pole to pole. The early Atlantic ocean provides an example. Suppose the ocean's widths are all less than one quarter the planet's circumference. In that case, the response to the atmospheric pressure loading maximum will be that water will move north and south away from the equator, since the atmospheric pressure is high near the equator. Water underneath the atmospheric pressure minimum will move toward the equator. In neither case will the total mass between a pair of meridians change, although the mean moment arm will be reduced (for a pressure maximum) or enhanced (for a minimum) as water moves towards or away from the poles. The total (air plus water) mass quadruple will be reduced, but not eliminated.

To account for the possibility that the ocean might act like an inverse barometer, we allow $A(t)$ in eqn. (**S32**) to be less than unity; for most Monte Carlo runs we choose a lower limit of 0.1. The best fit model from our MCMC calculations is shown in Fig. 4 in the main text.

The fixed LOD ≈ 19.5 hours implied by the data, as late as $1,300$ Ma, requires a thermal torque that is much larger than that today, and hence a large $A_2 = 3.51$ (note that the median value from the MCMC calculation is somewhat larger, $A_2 = 3.58$, see Table 1). This declines toward the present, so that at $1,300$ Ma, $N(1,300) \approx 2.35$. At the current epoch, the best fit

$N(0) = A_1 = 0.17$. We point out in the main text that this low value might be a result of the assumption, made in current cyclostratigraphic work, that L_{EM} is constant.

S3 Atmospheric composition over time

There is good evidence that the composition of the atmosphere changed over geologic time; see for example, reference (*54*), in particular, their Figs. 2, 3, and 5. The oxygen content was about a factor of 100 lower during the Proterozoic than today, and about a factor of 10,000,000 lower in the Archean. With somewhat less confidence, the CO_2 content was about 100 times higher at the beginning of the Proterozoic, while the total mass of the atmosphere was probably similar to that today. Our dynamical modeling favors a short atmospheric resonant period, which implies a combination of a high mean surface temperature and a low mean molecular mass. Fig. 7 in the main text shows an example atmospheric composition history. The values of P_{O_2} (orange dashed line) and P_{CO_2} (solid black line) in that figure are similar to those in (*54*). The total mean surface pressure $\bar{P}_s$ in this example is 2 bar. We show our inferred P_{CO_2} for several epochs, $t_{\rm th} = 700$, $1,000$, $1,500$, $1,800$, and $2,000$ Ma, denoted by green squares with error bars. These CO_2 levels yield sound speeds, and hence resonant periods $P_{\rm res}(t)$ that agree with the values of $P_{\rm res}$ found in the Monte Carlo calculations we use to find a best fit dynamical model (see section 6, subsection “Resonant period versus age”, below, for more discussion).

S4 Global Circulation Models

We are interested in a description of how the semi-diurnal atmospheric tide responds to changes in atmospheric properties and solar luminosity over geological times. The thermal tide behaves roughly as a Lamb wave, see (*61*) or (*100*), in essence a vertically-global sound-wave. We go beyond previous idealized setups, which employ a static 1D atmosphere (*101*) and (2) by using GCMs to describe this atmospheric mode. This allows us to capture the effects of a moist atmospheric circulation in a 3D inhomogeneous atmosphere on the thermal tide forcing and response.

We utilize two well tested and complementary GCMs, PlaSim and LMD-G, and find that they exhibit consistent behaviours for the Lamb mode of interest here, with only minor quantitative discrepancies on the mode resonant frequency.

PlaSim is an intermediate-complexity Earth GCM that includes a slab ocean, sea ice, land surface hydrology, snow, and a coupled atmosphere that includes moist processes and a simple radiation scheme (*41*). PlaSim allows for a simplified topography; for most of our runs this option was turned off. We performed some runs to check whether the topography affected either the mode frequency or $Q_{\rm th}$, but the differences were very small, within the variation found by running for different lengths of time. PlaSim solves the primitive equations using a spectral core, i.e., they are solved in Fourier space; to maintain numerical stability, numerical hyperviscosity, of the form $\propto \nabla^8$, is applied. PlaSim is typically run with an effective spatial resolution

corresponding to 32 latitude cells by 64 longitude cells (denoted T21) or 64 latitude and 128 longitude cells (T42), with 5 or 10 vertical layers. Most of our runs used the T21 resolution with 10 vertical layers. The radiation scheme uses a two-band shortwave parameterization, with gray water and ozone absorption in the red and blue bands respectively, gray cloud scattering, and a single-band longwave radiation scheme with gray absorption from water, CO2, and clouds. PlaSim has been used to study modern Earth climate (*102–105*), paleoclimate and snowball climate dynamics (*106–109*), and tidally-locked and slow-rotating planets (*109–111*).

LMD-G (or LMD Generic) is a 3D GCM derived from the LMDz three-dimensional Earth (*112*) and Mars (*113*) GCMs. LDM-G solves the primitive equations using a finite difference method on an Arakawa C grid. LMD-G employs flexible radiative transfer based on the correlated-k method, (*114*), and thermodynamics/cloud microphysics prescriptions to simulate a broad range of atmospheric gas compositions. The code has been used in many climate studies of solar system planets (43, 44, 54) and (*115, 116*), and exoplanets in a wide range of conditions (*114, 117, 118*)

Fig. S5 shows the global mean temperature $\bar{T}$ of Earth calculated using LMD-G, as a function of age for several different atmospheric compositions. Consistent with previous work (*53, 119*), we find that, for a fixed composition, $\bar{T}$ increases with time; this increase is driven by the increase in solar luminosity. As discussed in the previous section, the composition of the atmosphere has changed rather dramatically over geologic time; in particular, the amount of CO_2 in the atmosphere has decreased. The short resonant period $P_{\rm res}$ we infer from the geologic data suggests that the decrease of P_{CO_2} may have started as late as $\approx 1,5000$ Mya, much later than was previously believed. Alternately, P_{CO_2} may have varied non-monotonically, with a peak around $1,500$ Mya.

The results we obtain for the thermal tide using these two GCMs are qualitatively consistent with previous 1D treatments of the thermal tide response, as a damped, driven Lamb mode, e.g., Figs. S2 and S4, with the notable exception of the value of $Q_{\rm th}$. The 1D models find $Q_{\rm th} \approx 40 - 100$, while both our GCM models return $Q_{\rm th} \approx 12$. We have investigated the nature of the thermal tidal mode dissipation, building on the understanding developed by (*61, 100*). Through a parameter exploration, we have established that the mode dissipation in PlaSim is only weakly affected by the magnitude of surface friction or hyperviscosity. The dissipation is somewhat more sensitive to model resolution (both horizontal and vertical), but only at the ten percent level, so that the strong dissipation in GCMs is robust. Understanding the origin of this (possibly numerical) overdissipation is left to future work. We note that the vertically-limited description in our GCM models, focused on the lower tropospheric models, and the impact of the upper boundary condition adopted in this class of models, could play a role, given that the Lamb mode perturbation velocity grows exponentially with height (*120*).

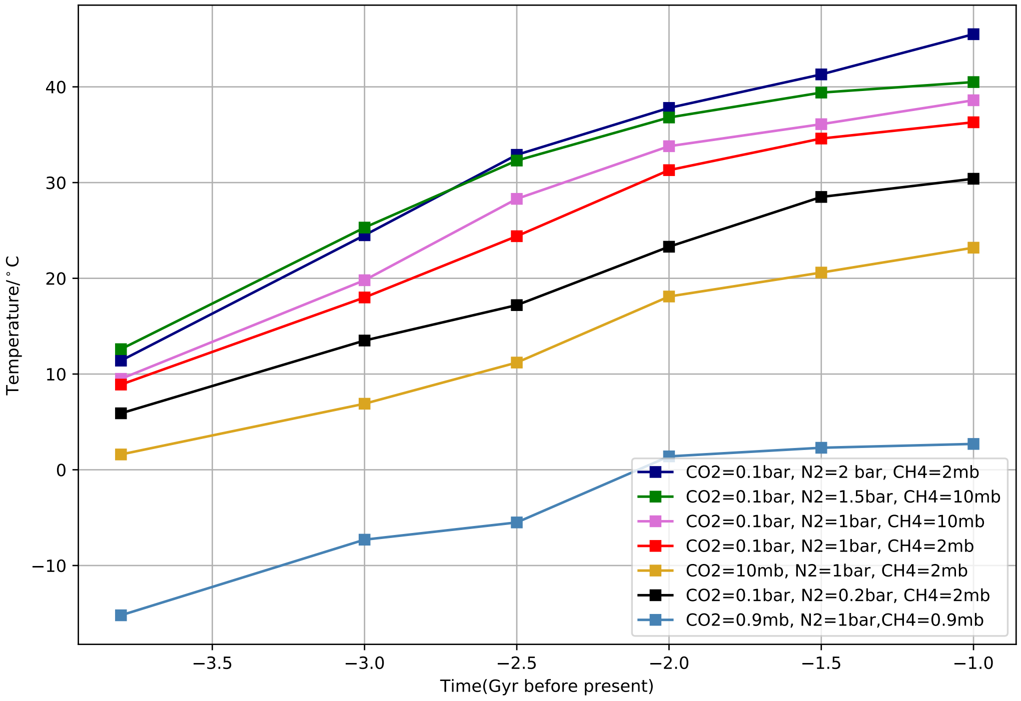

Fig. S5: The global mean temperature $\bar{T}$ as a function of age, for a number of different atmospheric compositions Z, as calculated by LMD-G. Different shaped markers connected by colored lines refer to different atmospheric compositions Z and surface pressures P_s, as labeled in the figure legend. The LOD is fixed at 24 hours, while the solar luminosity is assumed to vary with epoch as $L(t) = L_\odot / \left[1 + \frac{2}{5}(1 - t/t_\odot)\right]$ (*48*), where $t = 0$ corresponds to the time of formation of the solar system, $t_\odot = 4,560$ Ma. Note that the figure labels on the x-axis correspond to $\tau \equiv (t - t_\odot)$. For a given (Z, P_S), the global mean temperatures found by PlaSim are several (~ 9 C) degrees, or about three percent, higher than those returned by LMDZ for high values of P_{CO_2}, as can also be seen in Fig. 5 in the main text.

S5 Choosing the initial Lunar semimajor axis

A number of mechanisms operating early in the history of the Earth-Moon system could have altered L_{EM}. The evection resonance (*121, 122*) provides an example. The resonance occurs when the precession period of the Lunar apsidal axis matches the length of the year. The torque exerted by the Sun on the Lunar orbit transfers angular momentum from the Lunar orbit to the Earth's orbit around the Sun. The same torque may result in a limit cycle rather than libration around a fixed point (*123*); both result in a reduction of L_{EM}. The evection resonance occurs when $a_☾ \approx 5R_\oplus$. The change in L_{EM} can be as large as a factor of two, much larger than the change effected by the thermal tide. Dissipation of Lunar obliquity tides, associated with the variation of the Laplace plane with distance from Earth, can reduce L_{EM} by a similar amount (*124*); this mechanism is most effective for $a_☾ \lesssim 30R_\oplus$. Other mechanisms that have been proposed to alter L_{EM} include the scattering of roughly a Lunar mass worth of planetesimals (*125*), and interactions between the newly formed Moon and the remnants of the disk out of which it formed (*126*). The former occurs when $a_☾ \lesssim 30R_\oplus$, the latter when $a_☾ \lesssim 5 - 7R_\oplus$.

We have used $a_☾ = 20R_\oplus$ as our initial condition, since the time to reach this distance is short ($\approx 30\,Myr$) while the time to reach $30R_\oplus$ is about 150 Myr.

S6 Monte Carlo parameter estimates

If we pick a set of parameters and initial conditions for eqns. (3-5) in the main text, and then integrate, we can generate predictions for the number of months per year, the number of days per month, and the length of day, at any time in Earth's history. These predictions can then be compared to the geologic data $\mathbf{D} \equiv \{x_i, \sigma_i\}$, where x_i denotes the number of months per year, days per month, or hour per day, depending on the index i; the σ_i's are the associated errors. Thus we have a generative model (see, e.g., ref (*127*), section 2.3).

We use a Bayesian approach, combined with Monte Carlo calculations, to use the geologic data to infer the probability distribution functions for $L_{\rm EM}$, $\mathbf{P}_{\rm res}(\mathbf{t})$, and other quantities of interest.

We start by describing the parameter set (denoted by $\boldsymbol{\theta}$) sampled by the Monte Carlo calculations. We then describe the likelihood function, and finally the prior probabilities we use for each parameter.

There are a number of quantities, listed below, that need to be specified in order to perform the integration. Several of these quantities will be of broad interest, including the initial value of the angular momentum of the Earth-Moon system $L_{\rm EM}$, which will inform Lunar formation theories, the quality factor of the thermal tide $Q_{\rm th}$, and the resonant period of the Earth's atmosphere $P_{\rm res}(t)$.

Given $P_{\rm res}(t)$, we can use the relation between $P_{\rm res}$ and $\bar{T}$ from our GCMs, shown in Fig. 5 in the main text, to find $\bar{T}(t)$, which is of interest to climate modelers.

The initial conditions required by eqns. (3-5) (in the main text) are $L_\oplus$, $S_\oplus$, and $L_{☾}$. The start time of the integration (about 30 Myrs later than the formation time) $\tau_{☾}$ of the Moon, also needs to be specified. We treat $L_\oplus(\tau_{☾})$ and $L_{☾}(\tau_{☾})$ as fixed numbers. We treat $L_{EM}(\tau_{☾}) = S_\oplus(\tau_{☾}) + L_{☾}(\tau_{☾})$ and $\tau_{☾}$ as parameters whose probability distribution functions we wish to find, using radio-isotope dates to constrain $\tau_{☾}$.

To evaluate the gravitational tidal torque using eqn. (**S1**), we need the function $Q(\omega^t)$, for which we use the model of (*6*), shown in Fig. S1. We introduce a dimensionless number Q_1 to scale the tidal torque, a freedom needed to meet the constraints on the formation time of the Moon.

To evaluate the thermal torque $T_{\rm th}$ using eqn. (**S31**), we need to specify the thermal torque normalization $A(t)$, the atmospheric resonant period $P_{\rm res}(t)$, and the thermal resonance quality factor $Q_{\rm th}$. As described above, we use the piece-wise model given in eqn. (**S32**) for $A(t)$, thereby introducing two parameters A_1 and A_2 into our Monte Carlo modeling. Equation (**S32**) also introduces t_0, but we regard this as a fixed parameter (we use $t_0 = 2,000$ Ma), and do not include it in the Monte Carlo parameter set. As noted in the main text, the quantity $Q_{\rm th}$ may well vary with time, but for simplicity we ignore any time dependence.

We use a piece-wise linear model for $P_{\rm res}(t)$; we choose a vector $\mathbf{P}_{\rm res} = (P_{\rm res,1}, \ldots, P_{\rm res,n_{th}})$ specifying the value of the resonant period of the Earth's atmosphere at various epochs. The length of this vector is denoted by $n_{\rm th}$, and chosen by the user, as is the corresponding vector of epochs $t_{\rm th,i}$ at which to specify $P_{\rm res}$. For the plots presented here, we use $n_{\rm th} = 10$, with epochs given by $\mathbf{t}_{\rm th} = (0, 0.7, 1.0, 1.5, 1.8, 2.0, 2.2, 2.5, 3.0, 4.6)$ Ga. Neither $n_{\rm th}$ nor $t_{\rm th,i}$ are treated as parameters for the purposes of the Monte Carlo calculation.

To summarize, the parameter set $\boldsymbol{\theta}$ we utilize for our Monte Carlo calculations includes the initial value of L_{EM}, the start time of the integration (related to the Lunar age) $\tau_{☾}$, the normalization of the Ocean tide model quality factor, denoted Q_1, the quality factor $Q_{\rm th}$ of the atmospheric resonance, the two parameters A_1, A_2 in eqn. (**S32**), and $\mathbf{P}_{\rm res}$.

We regularize the parameter set by taking the natural logarithm of each parameter, so that

$$\boldsymbol{\theta} = \ln\left(L_{EM}, \tau_{☾}, Q_1, Q_{\rm th}, A_1, A_2, \mathbf{P}_{\rm res}\right). \qquad \textbf{(S33)}$$

As part of the regularization, we specify L_{EM} in units of Earth's breakup angular momentum $L_s \equiv C\sqrt{GM_\oplus/R_\oplus^3}$ (where C is the largest moment of inertia of Earth, see table 1), the age $\tau_{☾}$ in Myr, and $\mathbf{P}_{\rm res}$ in hours.

Given this parameter set, we integrate eqns. (3-5). We then interpolate to the epochs where we have data, and calculate the appropriate observable. Next, we calculate the χ^2 of the model given the data values x_i and errors σ_i. Assuming that the data points are independent of each other, we employ the likelihood

$$\mathcal{L} \equiv P(\mathbf{D}|\boldsymbol{\theta}) = \Pi_i \frac{1}{\sqrt{2\pi\sigma_i}} \exp\frac{(x_i - X_i(\boldsymbol{\theta}))^2}{2\sigma_i^2}, \qquad \textbf{(S34)}$$

where $X_i(\boldsymbol{\theta})$ is the prediction of the model for the ith data point.

We use the Monte Carlo code emcee (*62*) to sample the posterior probability density function $P(\boldsymbol{\theta}|\mathbf{D})$ of the parameters $\boldsymbol{\theta}$, given the data $\mathbf{D}$. The posterior probability is related to the data and the predictions of eqns. (3-5), by Bayes' theorem

$$P(\boldsymbol{\theta}|\mathbf{D}) = \frac{P(\mathbf{D}|\boldsymbol{\theta})P(\boldsymbol{\theta})}{P(\mathbf{D})}. \quad \textbf{(S35)}$$

The probability $P(\boldsymbol{\theta})$ is called the prior, and needs to be specified. We take the denominator $P(\mathbf{D})$, often called the evidence, to be a normalization constant, and ignore it.

We use truncated Gaussian priors for all variables except A_1 and A_2, for which we use truncated Rayleigh distributions. The priors for the first six parameters are forced to be zero outside the following limits: the initial angular momentum of the Earth-Moon system satisfies $0.33 \leq L_{EM}/L_s \leq 0.37$, the integration start time time $4,530 \leq \tau_{☾}/Ma \leq 4,300$, the tidal Q normalization $0.5 \leq Q_1 \leq 2$, the atmospheric resonance quality factor $10. \leq Q_{\rm th} \leq 120$, while the torque normalization constants are in the ranges $0.1 \leq A_1 \leq 1.5$, and $1 \leq A_2 \leq 10$.

We use Gaussian priors for $\mathbf{P}_{th}$ with mean $\mu = 22.8$ hr, except for the present epoch, for which we use 22.9 hr. The Gaussian widths $\sigma = 0.3$ hr, except for those at the epoch of the Minoan/Sturtian glaciations (700 Ma) and the Huronian glaciations (at $\approx 2,300$ Ma), for which $\sigma = 0.1$ hr; we discuss this further below. We have also explored priors with shorter resonant periods between 1000 Ma and 2000 Ma, roughly the "boring billion", but we don't present the results here, except to say that they are similar to our default case. The Gaussians are truncated (set to zero) outside (18.5, 23.5) hours, except for the present epoch, zeroed outside (22., 23.5) hours, and the previous epoch (at 700 Ma) which is zeroed outside the interval (18.0, 23.5) hours.

S6.1 Posterior parameter distributions, median values and ranges

Fig. S6 shows a corner plot for our full thermal plus gravitational tide model. It consists of all the pair-wise joint posterior parameter distributions of the model, as well as the single-parameter posterior distributions (along the hypotenuse of the triangle). Points in the joint distributions are samples from the Markov chain from emcee, with contours at 0.5, 1, 1.5, and 2σ.

Figure 6 reveals a strong (anti) correlation between L_{EM}/L_s and Q_1; small values of L_{EM}/L_s are associated with large values of Q_1. Our integrations start with a fixed value of $L_{☾}$, and effectively end with the present day value, so $\Delta L_{☾}$, which equals the integrated Lunar torque, is very nearly fixed, independent of $\boldsymbol{\theta}$. The Figure shows that the correlation between Q_1 and $\tau_{☾}$ is weak, so varying Q_1 does not greatly affect the time over which we integrate. However, the spin frequency of Earth strongly affects the Lunar tidal torque, as can be inferred from Fig. S1; higher spin frequencies produce higher tidal Q, and hence lower tidal torques. Very roughly we have $Q(\omega^t) \sim \omega_{\oplus} \sim S_{\oplus} \sim L_{EM}$. The spin frequency of Earth (or the inverse LOD) is strongly affected by varying L_{EM}, since we fix the initial $a_{☾} = 30R_{\oplus}$. This yields $L_{☾} = 1.66 \times 10^{41}\, dyne\, cm$ and $S_{\oplus} = 1.64 \times 10^{41}\, dyne\, cm$. An increase in $L_{EM}(\tau_{☾})$ at fixed $L_{☾}$ increases the initial $S_{\oplus}$, the initial tidal frequency, and hence the initial Lunar tidal torque.

Table S2: Monte Carlo full physics model median parameter values.

L_{EM}/L_s	$0.332^{0.00087}_{-0.00088}$
$\tau_{☾}\ (Ma)$	4545.68^{10}_{-15}
Q_1	$1.11^{0.024}_{-0.023}$
$Q_{\rm th}$	$100.71^{7.2}_{-7.5}$
A_1	$0.10^{0.059}_{-0.034}$
A_2	$3.58^{0.33}_{-0.31}$
$P_{\rm res,1}$ (hr)	$22.03^{0.043}_{-0.02}$
$P_{\rm res,2}$ (hr)	$22.12^{0.072}_{-0.067}$
$P_{\rm res,3}$ (hr)	$20.50^{0.18}_{-0.19}$
$P_{\rm res,4}$ (hr)	$18.66^{0.12}_{-0.097}$
$P_{\rm res,5}$ (hr)	$20.16^{0.17}_{-0.18}$
$P_{\rm res,6}$ (hr)	$21.66^{0.18}_{-0.18}$
$P_{\rm res,7}$ (hr)	$22.76^{0.1}_{-0.1}$
$P_{\rm res,8}$ (hr)	$22.82^{0.29}_{-0.29}$
$P_{\rm res,9}$ (hr)	$22.81^{0.29}_{-0.29}$
$P_{\rm res,10}$ (hr)	$22.79^{0.3}_{-0.3}$

Note–Estimated values are the medians of the posterior distributions. The uncertainties give the 16th and 84th percentiles. The epochs corresponding to P_1-P_{10} are $\mathbf{t}_{\rm th} = (0., 0.7, 1.0, 1.5, 1.8, 2.0, 2.2, 2.5, 3.0, 4.6)$ Ga.

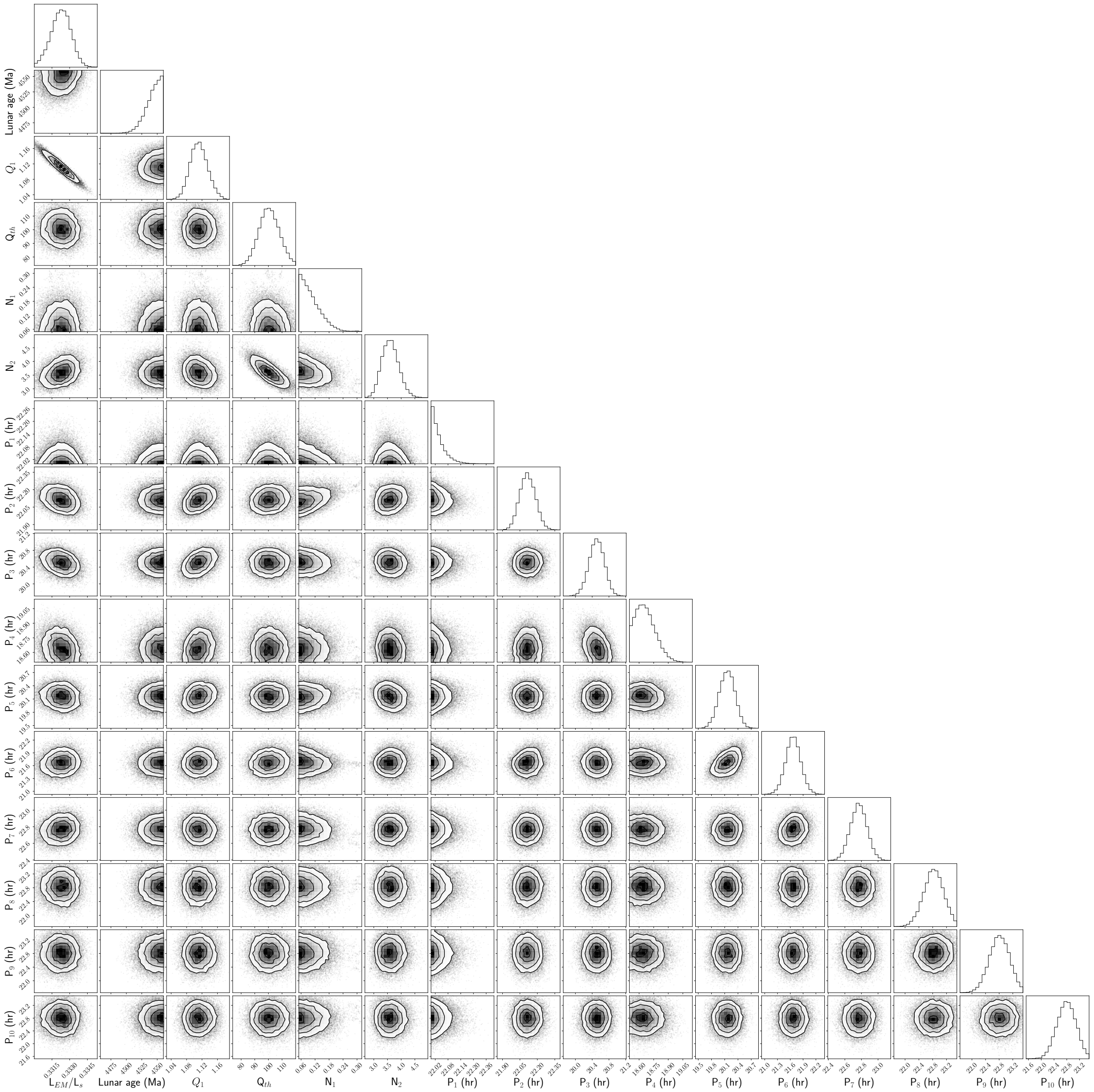

Fig. S6: Corner plot for the thermal tide model (*128*). There is a clear anti-correlation between L_{EM} and the normalization of the ocean tide model: lower values of L_{EM} are associated with higher values of Q_1 (and Q) and hence lower gravitational tidal dissipation rates. There is also a strong anti-correlation between $Q_{\rm th}$ and the maximum thermal torque normalization A_2; this is consistent with the notion that the thermal torque has to exceed some critical value (associated with $T_☾$ at the time of resonance capture). Table 2 gives the median values and error estimates (corresponding to the 16th and 84th percentile values) for all parameters.

The data, in the form of the present day LOD, fix the final value of the tidal frequency. It follows that an increase in L_{EM}, by producing an increase ω^t and hence $Q(\omega^t)$, forces a decrease in Q_1, the normalization of $T_{☾}$:

$$\Delta L_{☾} \sim \frac{1}{Q_1} \int dt\, a_{☾}^6(t)/Q(\omega^t(t)) \sim \frac{1}{Q_1 \omega_\oplus} \approx const., \tag{S36}$$

where we approximate $\omega^t \sim 2\omega_\oplus$. Recall that $L_{EM} = S_\oplus + L_{☾}$, so $\omega_\oplus \sim L_{EM} - L_{☾}$. It follows that

$$Q_1 \sim \frac{1}{\omega_\oplus} \sim \frac{1}{L_{EM} - L_{☾}}. \tag{S37}$$

Fig. S6 also reveals a fairly strong anti-correlation between $Q_{\rm th}$ and A_2. Increasing $Q_{\rm th}$ increases the peak $T_{\rm th}$, as does increasing A_2, so the peak $T_{\rm th}$ increases with the product $Q_{\rm th}A_2$. The peak value of $T_{\rm th}$ has to exceed $T_{☾}$ around $1,500$ Ma if the LOD is to be constant or decreasing. Hence, $Q_{\rm th}A_2 > const. \times T_{☾}$, leading to the inverse correlation we find.

Table 2 gives the median parameter values and the values corresponding to the 16th and 84th percentiles of the distribution functions (the super- and subscripts on the median values).

S6.2 Resonant period and mean temperature versus age

In the main text we use the posteriors for $\mathbf{P}_{\rm res}$ to infer the global mean temperature as a function of geologic age. Figure S7 shows the posteriors for $P_{\rm res}$ as a function of age (the median is given by the blue line; the shading indicates the 16th and 84th percentiles). The figure also shows the means and standard deviations of the gaussian priors for $P_{\rm res}$ (the points and error bars); the values of the epochs $t_{\rm th,i}$ are given in the figure caption.

The best fit posterior mean, together with the 16th and 84th percentiles, are listed in Table 2, where P_1 corresponds to the resonant period at present, and P_{10} corresponds to the resonant period at the formation time of the solar system.

We note that P_{10} is unconstrained by the data, so it is set by our choice of prior. In fact, the data do not constrain $P_{\rm res}$ for ages older than $2,500$ Ma, as indicated by the agreement between the priors and the posterior at those early epochs. This is expected, since the length of day was 17.5 hours or less, shorter than any plausible $P_{\rm res}$. Under these conditions, the thermal torque is non-resonant and thus small, so that it leaves a signature on the spin evolution that is not detectable using our technique.

The low value of $P_{\rm res}$ we infer around $1,500$ Mya is driven by the length of day between $2,000$ Ma and $1,400$ Ma. We note that the strongest constraint is that given by the short LOD (and small error bars) measured by reference (*17*); we have already noted that this data point relies on the assumption that L_{EM} is constant, an assumption which our results call into question.

Since we know the dependence of $P_{\rm res}(\bar{T})$ on the global mean temperature, we can invert that relation to find the global mean $\bar{T}$ as a function of age. This is how we convert the results for $P_{\rm res}$ as a function of age, shown in Fig. S7, to the run of mean global temperature with age shown in Fig. 7 in the main text.

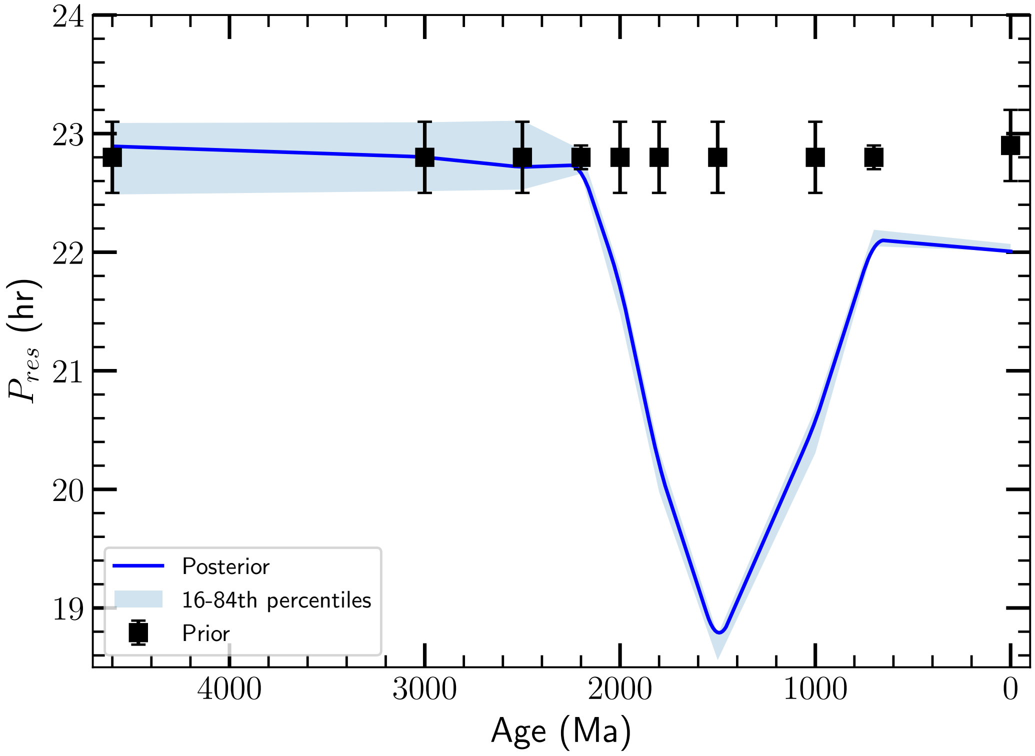

Fig. S7: The period of the atmospheric resonance P_{res} versus age, as inferred from our Monte Carlo calculation. The period is estimated at ten epochs, with ages of $\mathbf{t}_{\mathrm{res}} = (0., 0.7, 1.0, 1.5, 1.8, 2.0, 2.2, 2.5, 3.0, 4.6)$ Ga; the epochs at 0.7 and 2.2 Ga are chosen to allow for priors at the ages of known global scale glaciations (the Minoan/Sturtian at 0.7 and the Huronian at $\approx 2.2 - 2.5$ Ga). We use Gaussian priors with a mean of $\mu_i = 22.8$ hr, except for the present, for which the mean is $\mu_i = 22.9$ hr. The standard deviations are set to $\sigma_i = 0.3$ hr, except for the two glacial epochs, for which we choose a standard deviation of $\sigma_i = 0.1$ hr, to ensure that $\bar{T}$ is low enough to allow for global glaciations. The black squares and error bars show the mean and standard deviations for our priors. The blue solid line shows the mean P_{res}, while the blue shading shows the 16th and 84th percentile (correspond to one sigma for Gaussian errors) values for the inferred P_{th}. For ages between ≈ 2.0 Ga and ≈ 1 Ga, the data strongly favors $P_{\mathrm{res}} \approx 19-21$ h. For more recent epochs, the data is consistent with $P_{\mathrm{res}} \approx 22.0$ hr, about an hour shorter than at present. For ages older than ≈ 2.5 Ga, the percentile ranges correspond roughly to the standard deviations of the prior, indicating that the data does not provide strong constraints on P_{res} at these epochs.

Using the posteriors for $P_{\rm res}$ for epochs between 700 and $2,000$ Ma, we can infer the corresponding P_{CO_2} levels using Fig. 5 in the main text; we use the PlaSim results for each epoch, interpolating between the simulated values of $P_{CO_2} = [1, 10, 100, 200, 300]$ mbar. The results are shown as the green squares with error bars in Fig. 8 in the main text. Our inferred values of P_{CO_2} are at or above the 95% confidence level reported in (*54*). However, we note that there are no data points reported or referenced in that paper between $1,200$ and $1,800$ Ma.

REFERENCES AND NOTES

1. W. Thomson, 2. On the thermodynamic acceleration of the Earth's rotation. *Proc. R. Soc. Edinb.* **11**, 396–405 (1882).

2. K. Zahnle, J. C. G. Walker, A constant daylength during the precambrian era? *Precambrian Res.* **37**, 95–105 (1987).

3. J. A. M. Green, M. Huber, D. Waltham, J. Buzan, M. Wells, Explicitly modelled deep-time tidal dissipation and its implication for lunar history. *Earth Planet. Sci. Lett.* **461**, 46–53 (2017).

4. H. Daher, B. K. Arbic, J. G. Williams, J. K. Ansong, D. H. Boggs, M. Müller, M. Schindelegger, J. Austermann, B. D. Cornuelle, E. B. Crawford, O. B. Fringer, H. C. P. Lau, S. J. Lock, A. C. Maloof, D. Menemenlis, J. X. Mitrovica, J. A. M. Green, M. Huber, Long-term Earth-Moon evolution with high-level orbit and ocean tide models. *J. Geophys. Res.(Planets)* **126**, e2021JE006875 (2021).

5. M. Farhat, P. Auclair-Desrotour, G. Boué, J. Laskar, The resonant tidal evolution of the Earth-Moon distance. *Astron. Astroph.* **665**, L1 (2022).

6. D. J. Webb, Tides and the evolution of the Earth–Moon system. *Geophys. J.* **70**, 261–271 (1982).

7. T. Sakazaki, K. Hamilton, An array of ringing global free modes discovered in tropical surface pressure data. *J. Atmos. Sci.* **77**, 2519–2539 (2020).

8. W. B. N. Berry, R. M. Barker, Fossil bivalve shells indicate longer month and year in cretaceous than present. *Nature* **217**, 938–939 (1968).

9. G. Pannella, in *14th International Geological Congress* (Montréal, Canada, 1972), pp. 50–57.

10. G. Pannella, Paleontological evidence on the Earth's rotational history since early precambrian. *Astroph. Sp. Sci.* **16**, 212–237 (1972).

11. C. P. Sonett, A. Zakharian, E. P. Kvale, Ancient tides and length of day: Correction. *Science* **274**, 1068–1069 (1996).

12. G. E. Williams, Precambrian length of day and the validity of tidal rhythmite paleotidal values. *Geophys. Res. Lett.* **24**, 421–424 (1997).

13. C. P. Sonett, M. A. Chan, Neoproterozoic Earth-Moon dynamics: Rework of the 900 Ma Big Cottonwood Canyon tidal laminae. *Geophys. Res. Lett.* **25**, 539–542 (1998).

14. J. C. G. Walker, K. J. Zahnle, Lunar nodal tide and distance to the Moon during the Precambrian. *Nature* **320**, 600–602 (1986).

15. G. E. Williams, Geological constraints on the Precambrian history of Earth's rotation and the Moon's orbit. *Rev. Geophys.* **38**, 37–59 (2000).

16. L. A. Hinnov, Cyclostratigraphy and its revolutionizing applications in the Earth and planetary sciences. *Geol. Soc. Am. Bull.* **125**, 1703–1734 (2013).
17. S. R. Meyers, A. Malinverno, Proterozoic Milankovitch cycles and the history of the solar system. *Proc. Natl. Acad. Sci. U.S.A.* **115**, 6363 (2018).
18. M. Milankovitch, *Kanon der Erdbestrahlung und seine Anwendung auf das Eiszeitenproblem* (Mihaila Curcica, 1940).
19. J. D. Hays, J. Imbrie, N. J. Shackleton, Variations in the Earth's orbit: Pacemaker of the ice ages. *Science* **194**, 1121–1132 (1976).
20. C. Wunsch, Quantitative estimate of the Milankovitch-forced contribution to observed quaternary climate change. *Quat. Sci. Rev.* **23**, 1001–1012 (2004).
21. J. Laskar, P. Robutel, F. Joutel, M. Gastineau, A. C. M. Correia, B. Levrard, A long-term numerical solution for the insolation quantities of the Earth. *Astron. Astroph.* **428**, 261–285 (2004).
22. J. Laskar, A. Fienga, M. Gastineau, H. Manche, La2010: A new orbital solution for the long-term motion of the Earth. *Astron. Astroph.* **532**, A89 (2011).
23. M. D. Norman, L. E. Borg, L. E. Nyquist, D. D. Bogard, Chronology, geochemistry, and petrology of a ferroan noritic anorthosite clast from Descartes breccia 67215: Clues to the age, origin, structure, and impact history of the lunar crust. *Meteorit. Planet. Sci.* **38**, 645–661 (2003).
24. M. Barboni, P. Boehnke, B. Keller, I. E. Kohl, B. Schoene, E. D. Young, K. D. McKeegan, Early formation of the Moon 4.51 billion years ago. *Sci. Adv.* **3**, e1602365 (2017).
25. K. A. Eriksson, E. L. Simpson, Quantifying the oldest tidal record: The 3.2 Ga Moodies Group, Barberton Greenstone Belt, South Africa. *Geology* **28**, 831–834 (2000).
26. R. Mazumder, Implications of lunar orbital periodicity from the Chaibasa tidal rhythmite (India) of late Paleoproterozoic age. *Geology* **32**, 841–844 (2004).
27. H. N. Bhattacharya, B. Bhattacharya, S. Pal, A. Roy, Late Archaean tidalites from western margin of Chitradurga greenstone belt, southern India. *Precambrian Res.* **257**, 109–113 (2015).
28. C. T. Scrutton, Periodicity in devonian coral growth. *Palaeontology* **7**, 552–558 (1964).
29. S. J. Mazzullo, Length of the year during the silurian and devonian periods: New values. *Geol. Soc. Am. Bull.* **82**, 1085–1086 (1971).
30. R. E. Mohr, in *Growth Rhythms and the History of the Earth's Rotation*, G. D. Rosenberg, S. K. Runcorn, Eds. (John Wiley & Sons, Univ. California, 1975), vol. 12, pp. 43–56.

31. W. B. Berry, R. M. Barker, in *Growth Rhythms and the History of the Earth's Rotation*, G. D. Rosenberg, S. K. Runcorn, Eds. (Wiley, 1975), vol. 12, pp. 162–171.

32. G. A. L. Johnson, J. R. Nudds, in *Growth Rhythms and the History of Earth's Rotation*, G. D. Rosenberg, S. K. Runcorn, Eds. (Wiley, 1975), pp. 27–42.

33. J. W. Wells, Coral growth and geochronometry. *Nature* **197**, 948–950 (1963).

34. J. P. Vanyo, S. M. Awramik, Length of day and obliquity of the ecliptic 850 MA ago: Preliminary results of a stromatolite growth model. *Geophys. Res. Lett.* **9**, 1125–1128 (1982).

35. G. J. F. MacDonald, Tidal friction. *Rev. Geophys. Space Phys.* **2**, 467–541 (1964).

36. P. Goldreich, S. Soter, Q in the solar system. *Icarus* **5**, 375–389 (1966).

37. K. Lambeck, The Earth's variable rotation: Some geophysical causes, in *The Earth's Variable Rotation: Geophysical Causes and Consequences* (Cambridge Univ. Press, 1980), vol. 119, pp. 219-220.

38. J. O. Dickey, P. L. Bender, J. E. Faller, X. X. Newhall, R. L. Ricklefs, J. G. Ries, P. J. Shelus, C. Veillet, A. L. Whipple, J. R. Wiant, J. G. Williams, C. F. Yoder, Lunar laser ranging: A continuing legacy of the apollo program. *Science* **265**, 482–490 (1994).

39. S. Chapman, R. Lindzen, *Atmospheric Tides. Thermal and Gravitational* (Gordon and Breach, 1969).

40. R. Strachan, The diurnal range of atmospheric pressure. *Q. J. Roy. Meteorol. Soc.* **6**, 42 (1880).

41. K. Fraedrich, H. Jansen, E. Kirk, U. Luksch, F. Lunkeit, The planet simulator: Towards a user friendly model. *Meteorol. Z.* **14**, 299–304 (2005).

42. F. Forget, R. Wordsworth, E. Millour, J. B. Madeleine, L. Kerber, J. Leconte, E. Marcq, R. M. Haberle, 3D modelling of the Early martian climate under a denser CO_2 atmosphere: Temperatures and CO_2 ice clouds. *Icarus* **222**, 81–99 (2013).

43. J. Leconte, F. Forget, B. Charnay, R. Wordsworth, A. Pottier, Increased insolation threshold for runaway greenhouse processes on Earth-like planets. *Nature* **504**, 268–271 (2013).

44. J. Leconte, H. Wu, K. Menou, N. Murray, Asynchronous rotation of Earth-mass planets in the habitable zone of lower-mass stars. *Science* **347**, 632–635 (2015).

45. C. F. Yoder, *Global Earth Physics: A Handbook of Physical Constants*, T. J. Ahrens, Ed. (American Geophysical Union, 1995), p. 1.

46. C. Covey, A. Dai, D. Marsh, R. S. Lindzen, The surface-pressure signature of atmospheric tides in modern climate models. *J. Atmos. Sci.* **68**, 495–514 (2011).

47. C. Covey, A. Dai, R. S. Lindzen, D. R. Marsh, Atmospheric tides in the latest generation of climate models. *J. Atmos. Sci.* **71**, 1905–1913 (2014).

48. D. O. Gough, Solar interior structure and luminosity variations. *Sol. Phys.* **74**, 21–34 (1981).
49. K. Zahnle, M. Claire, D. Catling, The loss of mass-independent fractionation in sulfur due to a palaeoproterozoic collapse of atmospheric methane. *Geobiology* **4**, 271–283 (2006).
50. N. J. Planavsky, C. T. Reinhard, T. T. Isson, K. Ozaki, P. W. Crockford, Large mass-independent oxygen isotope fractionations in mid-proterozoic sediments: Evidence for a low-oxygen atmosphere? *Astrobiology* **20**, 628–636 (2020).
51. H. Kienert, G. Feulner, V. Petoukhov, Faint young Sun problem more severe due to ice-albedo feedback and higher rotation rate of the early Earth. *Geophys. Res. Lett.* **39**, L23710 (2012).
52. E. T. Wolf, O. B. Toon, Hospitable archean climates simulated by a general circulation model. *Astrobiology* **13**, 656–673 (2013).
53. B. Charnay, F. Forget, R. Wordsworth, J. Leconte, E. Millour, F. Codron, A. Spiga, Exploring the faint young Sun problem and the possible climates of the Archean Earth with a 3-D GCM. *J. Geophys. Res. Atmos.* **118**, 10,414–10,431 (2013).
54. D. C. Catling, K. J. Zahnle, The Archean atmosphere. *Sci. Adv.* **6**, eaax1420 (2020).
55. R. C. Payne, D. Brownlee, J. F. Kasting, Oxidized micrometeorites suggest either high pCO_2 or low pN_2 during the Neoarchean. *Proc. Natl. Acad. Sci. U.S.A.* **117**, 1360–1366 (2020).
56. C. Goldblatt, M. W. Claire, T. M. Lenton, A. J. Matthews, A. J. Watson, K. J. Zahnle, Nitrogen-enhanced greenhouse warming on early Earth. *Nat. Geosci.* **2**, 891–896 (2009).
57. S. M. Som, D. C. Catling, J. P. Harnmeijer, P. M. Polivka, R. Buick, Air density 2.7 billion years ago limited to less than twice modern levels by fossil raindrop imprints. *Nature* **484**, 359–362 (2012).
58. S. M. Som, R. Buick, J. W. Hagadorn, T. S. Blake, J. M. Perreault, J. P. Harnmeijer, D. C. Catling, Earth's air pressure 2.7 billion years ago constrained to less than half of modern levels. *Nat. Geosci.* **9**, 448–451 (2016).
59. B. Marty, L. Zimmermann, M. Pujol, R. Burgess, P. Philippot, Nitrogen isotopic composition and density of the Archean atmosphere. *Science* **342**, 101–104 (2013).
60. B. Marty, G. Avice, D. V. Bekaert, M. W. Broadley, Salinity of the Archaean oceans from analysis of fluid inclusions in quartz. *C. R. Geosci.* **350**, 154–163 (2018).
61. R. S. Lindzen, D. Blake, Lamb waves in the presence of realistic distributions of temperature and dissipation. *J. Geophys. Res.* **77**, 2166–2176 (1972).
62. D. Foreman-Mackey, D. W. Hogg, D. Lang, J. Goodman, Emcee: The MCMC hammer. *Publ. Astron. Soc. Pac.* **125**, 306–312 (2013).

63. B. C. Bartlett, D. J. Stevenson, Analysis of a Precambrian resonance-stabilized day length. *Geophys. Res. Lett.* **43**, 5716–5724 (2016).
64. A. Bekker, A. Kaufman, J. Karhu, K. Eriksson, Evidence for Paleoproterozoic cap carbonates in North America. *Precambrian Res.* **137**, 167–206 (2005).
65. G. M. Young, Aspects of the Archean-Proterozoic transition: How the great Huronian Glacial Event was initiated by rift-related uplift and terminated at the rift-drift transition during break-up of Lauroscandia. *Earth Sci. Rev.* **190**, 171–189 (2019).
66. G. P. Halverson, P. F. Hoffman, D. P. Schrag, A. C. Maloof, A. H. N. Rice, Toward a Neoproterozoic composite carbon-isotope record. *Geol. Soc. Am. Bull.* **117**, 1181 (2005).
67. A. Bekker, A. J. Kaufman, J. A. Karhu, N. J. Beukes, Q. D. Swart, L. L. Coetzee, K. A. Eriksson, Chemostratigraphy of the paleoproterozoic duitschland formation, South Africa: Implications for coupled climate change and carbon cycling. *Am. J. Sci.* **301**, 261–285 (2001).
68. V. von Brunn, D. J. C. Gold, Diamictite in the Archaean Pongola sequence of southern Africa. *J. African Earth Sci.* **16**, 367–374.
69. G. M. Young, V. V. Brunn, D. J. C. Gold, W. E. L. Minter, Earth's oldest reported glaciation: Physical and chemical evidence from the Archean Mozaan Group (~2.9 Ga) of South Africa. *J. Geol.* **106**, 523–538 (1998).
70. B. N. Modie, A glacigenic interpretation of a neoarchaean (≈2.78 Ga) volcanogenic sedimentary sequence in the Nnywane formation, Sikwane, Southeast Botswana. *J. African Earth Sci.* **35**, 163–175 (2002).
71. J. Veizer, R. N. Clayton, R. W. Hinton, Geochemistry of Precambrian carbonates: IV. Early Paleoproterozoic (2.25 $\pm$ 0.25 Ga) seawater. *GeCoA* **56**, 875–885 (1992).
72. S. Kurucz, P. Fralick, M. Homann, S. Lalonde, Earth's first snowball event: Evidence from the early Paleoproterozoic Huronian Supergroup. *Precambrian Res.* **365**, 106408 (2021).
73. S. Sarangi, S. P. Mohanty, A. Barik, Rare earth element characteristics of Paleoproterozoic cap carbonates pertaining to the Sausar Group, Central India: Implications for ocean paleoredox conditions. *J. Asian Earth Sci.* **148**, 31–50 (2017).
74. P. F. Hoffman, D. P. Schrag, The snowball Earth hypothesis: Testing the limits of global change. *Terra Nova* **14**, 129–155 (2002).
75. P. F. Hoffman, Strange bedfellows: Glacial diamictite and cap carbonate from the Marinoan (635 Ma) glaciation in Namibia. *Sedimentology* **58**, 57–119 (2011).

76. R. T. Pierrehumbert, D. S. Abbot, A. Voigt, D. Koll, Climate of the Neoproterozoic. *Annu. Rev. Earth Planet. Sci.* **39**, 417–460 (2011).
77. G. E. Williams, Subglacial meltwater channels and glaciofluvial deposits in the Kimberley basin, Western Australia: 1.8 ga low-latitude glaciation coeval with continental assembly. *J. Geol. Soc. London* **162**, 111–124 (2005).
78. G. Kuipers, F. F. Beunk, F. M. van der Wateren, Periglacial evidence for a 1.91-1.89 Ga old glacial period at low latitude, Central Sweden. *Geol. Today* **29**, 218–221 (2013).
79. N. J. Geboy, A. J. Kaufman, R. J. Walker, A. Misi, T. F. de Oliviera, K. E. Miller, K. Azmy, B. Kendall, S. W. Poulton, Re-Os age constraints and new observations of Proterozoic glacial deposits in the Vazante group, Brazil. *Precambrian Res.* **238**, 199–213 (2013).
80. G. M. Young, Precambrian supercontinents, glaciations, atmospheric oxygenation, metazoan evolution and an impact that may have changed the second half of earth history. *Geosci. Front.* **4**, 247–261 (2013).
81. N. M. Roberts, The boring billion? - lid tectonics, continental growth and environmental change associated with the Columbia supercontinent. *Geosci. Front.* **4**, 681–691 (2013).
82. R. Buick, D. J. Des Marais, A. H. Knoll, Stable isotopic compositions of carbonates from the Mesoproterozoic Bangemall group, northwestern Australia. *Chem. Geol.* **123**, 153–171 (1995).
83. G. Shields, J. Veizer, Precambrian marine carbonate isotope database: Version 1.1. *Geochem. Geophys. Geosyst.* **3**, 1–12 (2002).
84. J. F. Kasting, Earth's early atmosphere. *Science* **259**, 920–926 (1993).
85. A. A. Pavlov, M. T. Hurtgen, J. F. Kasting, M. A. Arthur, Methane-rich Proterozoic atmosphere? *Geology* **31**, 87–90 (2003).
86. P. L. Corcoran, W. U. Mueller, E. H. Chown, Climatic and tectonic influences on fan deltas and wave- to tide-controlled shoreface deposits: Evidence from the Archaean Keskarrah formation, Slave Province, Canada. *Sediment. Geol.* **120**, 125–152 (1998).
87. W. U. Mueller, P. L. Corcoran, J. A. Donaldson, in *Sedimentology of a Tide- and Wave-Influenced High-Energy Archaean Coastline: The Jackson Lake Formation, Slave Province, Canada* (John Wiley & Sons Ltd., 2002), pp. 153–182.
88. W. Mueller, P. Corcoran, C. Pickett, Mesoarchean continental breakup: Evolution and inferences from the >2.8 Ga slave craton-cover succession, Canada. *J. Geol.* **113**, 23–45 (2005).
89. K. A. Eriksson, E. Simpson, Precambrian tidal facies, in *Principles of Tidal Sedimentology*, R. Davis Jr., R. Dalrymple, Eds. (Springer, 2012), pp. 397–419.

90. C. Wunsch, D. Stammer, Atmospheric loading and the oceanic “inverted barometer” effect. *Rev. Geophys.* **35**, 79–107 (1997).
91. G. D. Egbert, R. D. Ray, Significant dissipation of tidal energy in the deep ocean inferred from satellite altimeter data. *Nature* **405**, 775–778 (2000).
92. D. J. Webb, Tides and tidal friction in a hemispherical ocean centred at the equator. *Geophys. J.* **61**, 573–600 (1980).
93. S. G. Jennings, The mean free path in air. *J. Aerosol Sci.* **19**, 159–166 (1988).
94. G. L. Stephens, J. Li, M. Wild, C. A. Clayson, N. Loeb, S. Kato, T. L’Ecuyer, P. W. Stackhouse Jr., M. Lebsock, T. Andrews, An update on Earth’s energy balance in light of the latest global observations. *Nat. Geosci.* **5**, 691–696 (2012).
95. K. E. Trenberth, J. T. Fasullo, J. Kiehl, Earth’s global energy budget. *Bull. Am. Meteorol. Soc.* **90**, 311–324 (2009).
96. P. L. Read, J. Barstow, B. Charnay, S. Chelvaniththilan, P. G. J. Irwin, S. Knight, S. Lebonnois, S. R. Lewis, J. Mendonça, L. Montabone, Global energy budgets and ‘Trenberth diagrams’ for the climates of terrestrial and gas giant planets. *Q. J. Roy. Meteorol. Soc.* **142**, 703–720 (2016).
97. J. M. Wahr, The effects of the atmosphere and oceans on the Earth’s wobble -- I. Theory. *Geophys. J.* **70**, 349–372 (1982).
98. R. D. Ray, Precise comparisons of bottom-pressure and altimetric ocean tides. *J. Geophys. Res. Oceans* **118**, 4570–4584 (2013).
99. K. Balidakis, R. Sulzbach, L. Shihora, C. Dahle, R. Dill, H. Dobslaw, Atmospheric contributions to global ocean tides for satellite gravimetry. *J. Adv. Model. Earth Syst.* **14**, e2022MS003193 (2022).
100. R. S. Lindzen, D. Blake, Internal gravity waves in atmospheres with realistic dissipation and temperature part II. Thermal tides excited below the mesopause. *Geophys. Astrophys. Fluid Dyn.* **2**, 31–61 (1971).
101. R. Lindzen, Internal gravity waves in atmospheres with realistic dissipation and temperature part I. Mathematical development and propagation of waves into the thermosphere. *Geophys. Astrophys. Fluid Dyn.* **1**, 303–355 (1970).
102. S. C. Dekker, H. J. de Boer, V. Brovkin, K. Fraedrich, M. J. Wassen, M. Rietkerk, Biogeophysical feedbacks trigger shifts in the modelled vegetation-atmosphere system at multiple scales. *Biogeosciences* **7**, 1237–1245 (2010).

103. R. D. Garreaud, A. Molina, M. Farias, Andean uplift, ocean cooling and Atacama hyperaridity: A climate modeling perspective. *Earth Planet. Sci. Lett.* **292**, 39–50 (2010).

104. K. Haberkorn, C. Lemmen, R. Blender, K. Fraedrich, Iterative land proxy based reconstruction of SST for the simulation of terrestrial Holocene climate. *Earth Syst. Dyn. Discuss.* **3**, 149–200 (2012).

105. P. Nowajewski, M. Rojas, P. Rojo, S. Kimeswenger, Atmospheric dynamics and habitability range in Earth-like aquaplanets obliquity simulations. *Icarus* **305**, 84–90 (2018).

106. V. Lucarini, K. Fraedrich, F. Lunkeit, Thermodynamic analysis of snowball Earth hysteresis experiment: Efficiency, entropy production and irreversibility. *Q. J. Roy. Meteorol. Soc.* **136**, 2–11 (2010).

107. R. Boschi, V. Lucarini, S. Pascale, Bistability of the climate around the habitable zone: A thermodynamic investigation. *Icarus* **226**, 1724–1742 (2013).

108. M. Linsenmeier, S. Pascale, V. Lucarini, Climate of Earth-like planets with high obliquity and eccentric orbits: Implications for habitability conditions. *Planet. Space Sci.* **105**, 43–59 (2015).

109. J. H. Checlair, A. M. Salazar, A. Paradise, K. Menou, D. S. Abbot, No snowball cycles at the outer edge of the habitable zone for habitable tidally locked planets. *Ap. J. Lett.* **887**, L3 (2019).

110. J. Checlair, K. Menou, D. S. Abbot, No snowball on habitable tidally locked planets. *Ap. J.* **845**, 132 (2017).

111. D. S. Abbot, J. Bloch-Johnson, J. Checlair, N. X. Farahat, R. J. Graham, D. Plotkin, P. Popovic, F. Spaulding-Astudillo, Decrease in hysteresis of planetary climate for planets with long solar days. *Ap. J.* **854**, 3 (2018).

112. F. Hourdin, I. Musat, S. Bony, P. Braconnot, F. Codron, J. L. Dufresne, L. Fairhead, M. A. Filiberti, P. Friedlingstein, J. Y. Grandpeix, G. Krinner, P. LeVan, Z. X. Li, F. Lott, The LMDZ4 general circulation model: Climate performance and sensitivity to parametrized physics with emphasis on tropical convection. *Clim. Dynam.* **27**, 787–813 (2006).

113. F. Forget, F. Hourdin, R. Fournier, C. Hourdin, O. Talagrand, M. Collins, S. R. Lewis, P. L. Read, J. P. Huot, Improved general circulation models of the Martian atmosphere from the surface to above 80 km. *J. Geophys. Res.* **104**, 24155–24175 (1999).

114. R. Wordsworth, F. Forget, F. Selsis, E. Millour, B. Charnay, J.-B. Madeleine, Gliese 581d is the first discovered terrestrial-mass exoplanet in the habitable zone. *Ap. J. Lett.* **733**, L48 (2011).

115. R. Wordsworth, F. Forget, E. Millour, J. W. Head, J. B. Madeleine, B. Charnay, Global modelling of the early martian climate under a denser CO_2 atmosphere: Water cycle and ice evolution. *Icarus* **222**, 1–19 (2013).

116. M. Turbet, C. Gillmann, F. Forget, B. Baudin, A. Palumbo, J. Head, O. Karatekin, The environmental effects of very large bolide impacts on early Mars explored with a hierarchy of numerical models. *Icarus* **335**, 113419 (2020).

117. B. Charnay, V. Meadows, J. Leconte, 3D modeling of GJ1214b's atmosphere: Vertical mixing driven by an anti-hadley circulation. *Ap. J.* **813**, 15 (2015).

118. M. Turbet, J. Leconte, F. Selsis, E. Bolmont, F. Forget, I. Ribas, S. N. Raymond, G. Anglada-Escudé, The habitability of Proxima Centauri b. II. Possible climates and observability. *Astron. Astroph.* **596**, A112 (2016).

119. B. Charnay, G. Le Hir, F. Fluteau, F. Forget, D. C. Catling, A warm or a cold early Earth? New insights from a 3-D climate-carbon model. *Earth Planet. Sci. Lett.* **474**, 97–109 (2017).

120. G. K. Vallis, *Atmospheric and Oceanic Fluid Dynamics* (Cambridge Univ. Press, 2012).

121. W. M. Kaula, C. F. Yoder, in *Lunar and Planetary Science Conference* (1976), vol. 7, p. 440.

122. J. Touma, J. Wisdom, Resonances in the early evolution of the Earth-Moon system. *Astron. J.* **115**, 1653–1663 (1998).

123. J. Wisdom, Z. Tian, Early evolution of the Earth-Moon system with a fast-spinning Earth. *Icarus* **256**, 138–146 (2015).

124. M. Ćuk, D. P. Hamilton, S. J. Lock, S. T. Stewart, Tidal evolution of the Moon from a high-obliquity, high-angular-momentum Earth. *Nature* **539**, 402–406 (2016).

125. K. Pahlevan, A. Morbidelli, Collisionless encounters and the origin of the lunar inclination. *Nature* **527**, 492–494 (2015).

126. W. R. Ward, R. M. Canup, Origin of the Moon's orbital inclination from resonant disk interactions. *Nature* **403**, 741–743 (2000).

127. D. J. C. Mackay, *Information Theory, Inference and Learning Algorithms* (Cambridge Univ. Press, 2003).

128. D. Foreman-Mackey, Corner.Py: Scatterplot matrices in python. *J. Open Source Softw.* **1**, 24 (2016).
